# Regional and temporal variation in receipt of long‐term opioid therapy among older breast, colorectal, lung, and prostate cancer survivors in the United States

**DOI:** 10.1002/cam4.3709

**Published:** 2021-01-09

**Authors:** Derrick C. Gibson, Mukaila A. Raji, Jacques G. Baillargeon, Yong‐Fang Kuo

**Affiliations:** ^1^ Department of Preventive Medicine and Population Health University of Texas Medical Branch ‐ Galveston Galveston TX USA; ^2^ Division of Geriatrics and Palliative Medicine Department of Internal Medicine University of Texas Medical Branch ‐ Galveston Galveston TX USA; ^3^ Department of Preventive Medicine and Population Health Office of Biostatistics University of Texas Medical Branch ‐ Galveston Galveston TX USA

**Keywords:** analgesics, drug utilization, neoplasms, opioid, policy

## Abstract

**Background:**

Older cancer survivors have high rates of long‐term opioid therapy (≥90 days/year). However, the geographical and temporal variation in long‐term opioid therapy rates for older cancer survivors is not known.

**Methods:**

A retrospective cohort study was conducted using SEER‐Medicare data. Persons aged ≥66 years, diagnosed with breast, colorectal, lung, or prostate cancer from 1991 to 2011, and alive ≥5 years after diagnosis were included. Persons were followed from 1/1/2008 until 12/31/2016. Persons were assigned to a census region in their state of residence each year. Individuals who were covered by an opioid prescription for at least 90 days in a calendar year were classified as having received long‐term opioid therapy. Multivariable analysis was conducted using generalized estimating equations.

**Results:**

Temporal trends significantly varied by region (*p* < 0.0001) and opioid‐naïve status (*p* < 0.0001). Compared to 2013, opioid‐naïve cancer survivors in the south and non‐naïve survivors in the south and west experienced significant declines in long‐term opioid therapy in 2015 and 2016. Significant declines were observed in 2016 for opioid‐naïve and non‐naïve cancer survivors residing in the northeast and among opioid‐naïve cancer survivors living in the Midwest.

**Conclusion:**

The annual trends in the receipt of long‐term opioid therapy significantly varied by region among older cancer survivors. Variation in a clinical practice suggests the need for more research and interventions to improve efficiency, process, cost, and quality of care.

## Graphical abstract

Our study examines the regional and temporal trends in receipt of long‐term opioid therapy among older adults who have lived 5 or more years after cancer diagnosis. We found the annual trends in the receipt of long‐term opioid therapy were the highest in the south and lowest in the northeast. Variation in a clinical practice suggests the need for more research and interventions to improve efficiency, process, cost, and quality of care.

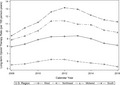

## 1 INTRODUCTION

Approximately 67% of persons diagnosed with cancer are expected to live at least 5 years after their cancer diagnoses.^1^ Chronic pain is common in patients and can last beyond the completion of cancer treatment.^2^, ^3^ Prescription opioids may be used to treat pain shortly after diagnosis, but a substantial number of older adults use prescription opioids years after a cancer diagnosis.^4^, ^5^ For example, Salz et al. (2019) found that older persons diagnosed with cancer were more likely to experience chronic opioid years after their diagnosis compared with persons without cancer, but this relationship varied with respect to the cancer diagnosis.^6^ Opioid therapy is associated with an increased risk of adverse events, such as falls and fractures,^7^ hypogonadism,^8^, ^9^ and heart disease,^10^ and utilization of prescription opioids for longer durations can increase the risk of adverse health outcomes particularly in older adults with a history of cancer.^11^

Previous studies have shown that opioid prescribing varies by time and geographical region in the United States.^12^, ^13^, ^14^, ^15^, ^16^ In general, opioid prescribing rates, amount of opioids dispensed, days supplied, and long‐term use are highest in Appalachia and the south. Furthermore, it appears prescription opioid prescribing peaked in 2010 and slowly declined until 2015.^15^, ^16^ Among older cancer survivors residing in Texas, Shah et al. (2019) observed that the prevalence of receipt of long‐term opioid therapy increased slowly from 2008 to 2010 but then increased sharply in 2011 and remained constant until 2014.^4^ Previous studies have provided significant insight into the geographical and temporal patterning of the utilization of prescription opioids, but national temporal and regional trends in receipt of long‐term opioid therapy have not been examined specifically for older adult cancer survivors.

The purpose of this study was to examine how annual rates in the receipt of long‐term opioid therapy changed across regions in the United States and by time for older persons with a history of breast, colorectal, lung, and prostate cancer diagnosis. We also examined whether the temporal trends varied by opioid naivety given that patterns of opioid use are influenced by previous opioid use among older cancer survivors.^4^ Understanding regional and temporal variations in long‐term opioid therapy is important given the dissemination of opioid prescribing guidelines and implementation of state and federal policies that sought to regulate opioid prescribing over the previous decade.

## 2 METHODS

### 2.1 Data source

A retrospective cohort study was performed using linked Surveillance, Epidemiology, and End Results (SEER)‐Medicare data sets. SEER is a program of cancer registries that began submitting cancer‐related information in 1973 from states or regions within states. Medicare provides health coverage to approximately 96% of US citizens aged 65 years and older. Persons are also eligible if they have received Social Security Disability benefits for 24 months or been diagnosed with end‐stage renal disease (ESRD) or amyotrophic lateral sclerosis. Fee‐for‐service Medicare includes Parts A and B, which cover inpatient hospital stays or outpatient services, respectively. Medicare Part D provides coverage for outpatient prescription drugs. The University of Texas Medical Branch Institutional Review Board (IRB) approved this study.

### 2.2 Study cohort

Annual cohorts were constructed for each year of the study (2008–2016) using the same inclusion and exclusion criteria. Persons who were included were followed from 1 January to 31 December of a given year. Persons were eligible for inclusion in this study if they were diagnosed with a cancer of the breast, colorectum, lung, or prostate as their first cancer diagnosis anytime between 1 January 1991 and 31 December 2011. These four cancers were chosen because they are the most common cancers diagnosed in the United States. Persons were assigned an index date corresponding to at least 5 years post‐cancer diagnosis, the date of survivorship. Individuals diagnosed with cancer before 1 January 2003 were assigned a date of survivorship of 1 January 2008 because they had survived greater than 5 years after cancer diagnosis and therefore were available on the first date of study.

Persons were excluded from the study if they were: (1) diagnosed at autopsy or on a death certificate, (2) had an unknown month of diagnosis or birth month or year, or (3) had been diagnosed with a second primary cancer. For each annual cohort, persons were excluded from the analysis if they were: (1) younger than 66 years of age on 1 January of the corresponding year, (2) had not been diagnosed for at least 5 years or the date of survivorship was later than 1 January of corresponding year (eg, 1 February 20XX), (3) had non‐continuous Part A, B, and D enrollment or had enrollment in a Health Maintenance Organization (HMO) in the 12 months prior to 1 January of the corresponding year, (4) had a claim for hospice care, were deceased, or had received cancer treatment (Table S1) in the 12 months prior to 1 January of the corresponding year, or (5) had non‐continuous enrollment in Part A, B, and D or enrollment in an HMO during the 12 months of follow‐up. Persons who died or had a claim for hospice care during a given year were censored at that date and were included in the study if they lived until 1 April of the corresponding year. The sample flowchart is presented in Figure S1.

### 2.3 Prescription opioid outcomes (long‐term opioid therapy)

National Drug Codes from RedBook were used to identify dispensed opioid prescriptions from the PDE file. The *cumulative* number of calendar days a person possessed an opioid prescription in a calendar year, from 1 January to 31 December, was calculated. We assumed the prescription began on the date of dispensing and ended on the date of dispensing + days supplied − 1, accounting for the filled date as the first day of the opioid prescription. Persons who had an opioid prescription for ≥90 days in a calendar year were classified as having received long‐term opioid therapy.

### 2.4 Covariates

Time‐invariant covariates were gender (male, female), race‐ethnicity (non‐Hispanic White, non‐Hispanic Black, non‐Hispanic other, Hispanic), diagnosis cohort, cancer diagnosis (breast, colorectal, lung, prostate), and original reason for Medicare entitlement (age‐related enrollment or non–age‐related). Diagnosis cohort was the recategorization of the year of cancer diagnosis into 5 cohorts (1991–1994, 1995–1998, 1999–2002, 2003–2006, and 2007–2011). Time‐varying covariates were the years post‐cancer diagnosis, age at the beginning of a calendar year (66–74, 75–84, ≥85 years), metropolis status (metropolis, urban‐rural), census region (west, northeast, Midwest, south), Medicaid eligibility, Charlson's comorbidity score ≥1, depressive disorder, anxiety disorder, alcohol use disorder, drug use disorder, and opioid‐naïve status. Opioid‐naïve cancer survivors were persons who did not receive any prescription opioid in the previous 12 months prior to 1 January of a calendar year. Appropriate diagnostic and procedure ICD‐9CM and ICD‐10 codes were utilized to identify relevant Charlson's comorbidities and mental health disorders.^17^, ^18^

### 2.5 Statistical analysis

Means (standard deviations) and medians (Quartile 1, Quartile 3) were calculated for continuous variables, and frequencies (percentages) were calculated for categorical covariates. Descriptive analysis was conducted to examine the distribution of patient characteristics by calendar year to assess how the composition of the overall cohort changed over time. In order to assess crude regional differences in long‐term opioid therapy, we calculated prevalence rates for each calendar year by dividing the total number of persons who received long‐term opioid therapy by the total number of person‐years contributed for each calendar year. For each calendar year, a person could contribute a minimum of 0.3 person‐years to a maximum of 1.0 person‐year.

Multivariable analysis estimating the adjusted odds ratio (aOR) of receipt of long‐term opioid therapy within each calendar year was conducted utilizing generalized estimating equations (GEE)^19^ with a binomial distribution, logit link function, and an autoregressive (AR1) correlation structure to account for repeated measures of persons. An offset statement was included for the person‐years contributed to each calendar year. To assess whether time trends differed across U.S. regions and opioid‐naïve status, we performed statistical interactions between the calendar year and census region and opioid‐naïve status by including each individual interaction term into the main‐effects model. Since the statistical interactions between year and region and year and opioid‐naïve status were significant, we stratified the models by these variables to examine how the temporal trends in receipt of long‐term opioid therapy varied by region and opioid‐naïve status. In the stratified analysis of opioid non‐naïve persons, the working correlation structure was specified as independent due to the non‐convergence of the AR1 model for this subgroup. We chose 2013 as the reference year because this was the year before the enforcement of the federal rescheduling of hydrocodone.^20^ All statistical tests were two‐sided with α = 0.05. All data management steps and analyses were performed with SAS version 9.4 (SAS Inc).

## 3 RESULTS

Overall, there were 344,443 persons who contributed a total of 1,255,333.8 person‐years. The minimum number of person‐years contributed by a single individual was 0.3 and the maximum was 9.0, with an average of 3.6 person‐years (Std = 2.5) and median of 3.0 person‐years (Q1, Q3 = 1.9, 5.0).

Table 1 and Table S2 demonstrate how the person characteristics changed at selected years during the study period. Overall, from 2008 to 2016, the sample became slightly younger. There were small increases in the percentage of persons diagnosed with depressive and anxiety disorders. From 2008 to 2016, there was a decline in the percentage of colorectal cancer survivors but an increase in the percentage of prostate cancer survivors. From 2008 to 2016, the percentage of northeastern and southern residents increased, while the percentage of midwestern and western residents decreased. However, the west comprised over 40% of the sample each year. One of the largest demographic changes during the study period was the composition of years of diagnosis. In 2008, most of the sample was comprised of persons diagnosed with cancer in 1999–2002 (59%). In 2016, no diagnosis cohort comprised a simple majority, but the 2007–2011 cohort comprised the largest percentage (35%). The 1991–1994 cohort and 1995–1998 cohort comprised 16% and 23% of the 2008 sample, respectively, but comprised 5% and 8% of the 2016 sample.

**TABLE 1:** Descriptive characteristics of older cancer survivors within each calendar year

	2008 (n = 74773)	2011 (n = 111243)	2014 (n = 190539)	2016 (n = 226417)
Variables				
Age mean (std)	78.4 (7.5)	78.3 (7.6)	78.0 (7.5)	77.8 (7.4)
Age median (IQR)	78.0 (72.2, 83.8)	77.5 (72.0, 83.8)	77.0 (71.8, 83.4)	76.9 (71.9, 83.1)
Age, categorical				
66–74 years	24490 (32.9%)	38017 (34.4%)	67024 (35.6%)	83878 (36.1%)
75–84 years	28961 (38.9%)	40909 (37.1%)	69745 (37.0%)	85678 (36.9%)
≥85 years	24490 (32.9%)	38017 (34.4%)	51636 (27.4%)	62624 (27.0%)
Years post‐cancer diagnosis				
Mean (std)	9.0 (3.3)	10.0 (3.7)	11.0 (4.3)	11.7 (4.6)
Median (IQR)	7.8 (6.3, 11.4)	9.2 (7.0, 11.9)	10.3 (7.5, 13.3)	11.0 (7.9, 14.6)
Gender				
Female	40640 (54.5%)	59243 (53.7%)	95895 (50.9%)	117976 (50.8%)
Male	33868 (45.5%)	51127 (46.3%)	92510 (49.1%)	114204 (49.2%)
Race and ethnicity				
Hispanic	4507 (6.0%)	7214 (6.5%)	10081 (5.4%)	12076 (5.2%)
Non‐Hispanic Black	5084 (6.8%)	7326 (6.6%)	14159 (7.5%)	16249 (7.0%)
Non‐Hispanic Other	5197 (7.0%)	7603 (6.9%)	11373 (6.0%)	14276 (6.1%)
Non‐Hispanic White	59720 (80.2%)	88227 (79.9%)	152792 (81.1%)	189579 (81.7%)
Cancer diagnosis				
Breast cancer	29896 (40.1%)	44703 (40.5%)	75001 (39.8%)	94149 (40.6%)
Colorectal cancer	14364 (19.3%)	19812 (18.0%)	29718 (15.8%)	34043 (14.7%)
Lung cancer	2846 (3.8%)	4173 (3.8%)	6575 (3.5%)	8044 (3.5%)
Prostate cancer	27402 (36.8%)	41682 (37.8%)	77111 (40.9%)	95944 (41.3%)
Diagnosis cohort				
1991–1994	12199 (16.4%)	10810 (9.8%)	12120 (6.4%)	11555 (5.0%)
1995–1998	17436 (23.4%)	16227 (14.7%)	18850 (10.0%)	18683 (8.0%)
1999–2002	43678 (58.6%)	42734 (38.7%)	52060 (27.6%)	52431 (22.6%)
2003–2006	1195 (1.6%)	40599 (36.8%)	66531 (35.3%)	68367 (29.4%)
2007–2011			38844 (20.6%)	81144 (34.9%)
Census region				
Midwest	13891 (18.6%)	18148 (16.4%)	29482 (15.6%)	32554 (14.0%)
Northeast	12476 (16.7%)	19212 (17.4%)	38225 (20.3%)	49424 (21.3%)
South	14160 (19.0%)	23556 (21.3%)	40821 (21.7%)	51814 (22.3%)
West	33981 (45.6%)	49454 (44.8%)	79877 (42.4%)	98388 (42.4%)
Urban‐rural status				
Metropolis	60394 (81.1%)	91220 (82.6%)	159580 (84.7%)	197609 (85.1%)
Rural	1718 (2.3%)	2332 (2.1%)	3444 (1.8%)	4102 (1.8%)
Urban	12396 (16.6%)	16818 (15.2%)	25381 (13.5%)	30469 (13.1%)
Metropolis or not				
Rural or urban	14114 (18.9%)	19150 (17.4%)	28825 (15.3%)	34571 (14.9%)
Metropolis	60394 (81.1%)	91220 (82.6%)	159580 (84.7%)	197609 (85.1%)
Original reason for enrollment				
Age	68499 (91.9%)	100954 (91.5%)	172700 (91.7%)	213030 (91.8%)
Disability and end‐stage renal disease	26 (0.0%)	55 (0.0%)	102 (0.1%)	141 (0.1%)
Disability	5954 (8.0%)	9310 (8.4%)	15485 (8.2%)	18859 (8.1%)
End‐stage renal disease	29 (0.0%)	51 (0.0%)	118 (0.1%)	150 (0.1%)
Age‐related medicare enrollment				
Not age related	6009 (8.1%)	9416 (8.5%)	15705 (8.3%)	19150 (8.2%)
Age related	68499 (91.9%)	100954 (91.5%)	172700 (91.7%)	213030 (91.8%)
Medicaid eligibility	16578 (22.2%)	23357 (21.2%)	27683 (14.7%)	30114 (13.0%)
Charlson's comorbidity ≥1	49138 (65.9%)	73821 (66.9%)	123725 (65.7%)	152987 (65.9%)
Depressive disorder	7949 (10.7%)	12609 (11.4%)	23167 (12.3%)	31092 (13.4%)
Anxiety disorder	4877 (6.5%)	8950 (8.1%)	19419 (10.3%)	28640 (12.3%)
Alcohol use disorder	718 (1.0%)	1106 (1.0%)	2699 (1.4%)	4174 (1.8%)
Drug use disorder	395 (0.5%)	719 (0.7%)	1718 (0.9%)	3297 (1.4%)
Opioid naïve	53491 (71.8%)	76461 (69.3%)	128567 (68.2%)	161347 (69.5%)

**FIGURE 1:**
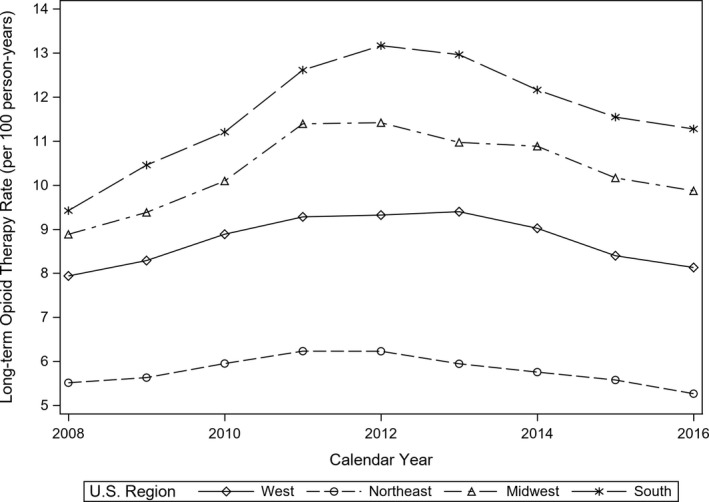
Rate of receipt of long‐term opioid therapy stratified by U.S. census region

Overall, the rate of long‐term opioid therapy increased from 8.0 persons with long‐term opioid therapy per 100 person‐years in 2008 to 10.0 in 2012 and then decreased to 8.5 in 2016. Figure 1 displays the rates of receipt of long‐term opioid therapy stratified by region. Throughout, the study period the south had the highest prevalence rates, and the northeast had the lowest rates. The rates of long‐term opioid therapy increased from 2008 to 2012 and declined from 2013 to 2016 across all regions. From 2008 to 2016, the rate of long‐term opioid therapy increased in the west from 7.9 per 100 person‐years (2008) to 8.1 (2016), increased in the Midwest from 8.9 (2008) to 9.9 (2016), and increased in the south from 9.4 (2008) to 11.3 (2016), but decreased in the northeast from 5.5 (2008) to 5.3 per 100 person‐years (2016).

After adjusting for patient demographics, cancer diagnosis, and comorbid conditions, the time trend in receipt of long‐term opioid therapy was found to vary significantly by U.S. region (*p* = 0.0002, not shown); therefore, we stratified our model assessing temporal trends of receipt of long‐term opioid therapy by census regions (Table 2). After 2013, there was no statistically significant decline in the trend of long‐term opioid therapy, overall, in the receipt of long‐term opioid therapy in the west, northeast, and Midwest. Instead, a statistically significant increase was observed in the Midwest in 2014 (aOR = 1.05, 95% CI: 1.00, 1.10). A significant decline was noted in the south in 2015 (aOR = 0.93, 95% CI: 0.89, 0.98) but not in 2014 (aOR = 0.97, 95% CI: 0.94, 1.01) and 2016 (aOR = 0.97, 95% CI: 0.91, 1.03).

**TABLE 2:** Adjusted odd ratios (aOR) and 95% confidence intervals of receipt of long‐term opioid therapy stratified by region

	West	Northeast	Midwest	South
Calendar year	aOR (95% CI)	aOR (95% CI)	aOR (95% CI)	aOR (95% CI)
2008	**0.89 (0.82, 0.98)**	1.04 (0.88, 1.24)	**0.86 (0.75, 0.98)**	**0.85 (0.77, 0.95)**
2009	**0.90 (0.84, 0.97)**	0.97 (0.84, 1.12)	**0.87 (0.78, 0.97)**	**0.88 (0.80, 0.96)**
2010	**0.94 (0.89, 1.00)**	0.97 (0.86, 1.09)	0.93 (0.85, 1.01)	**0.87 (0.80, 0.93)**
2011	0.98 (0.94, 1.03)	1.01 (0.92, 1.11)	1.06 (0.98, 1.13)	1.00 (0.94, 1.05)
2012	0.99 (0.96, 1.02)	0.98 (0.91, 1.04)	1.03 (0.98, 1.08)	0.99 (0.95, 1.03)
2013	REF	REF	REF	REF
2014	1.01 (0.98, 1.04)	0.98 (0.92, 1.04)	**1.05 (1.00, 1.10)**	0.97 (0.94, 1.01)
2015	0.96 (0.92, 1.00)	0.98 (0.91, 1.06)	0.99 (0.93, 1.06)	**0.93 (0.89, 0.98)**
2016	0.99 (0.93, 1.04)	1.02 (0.92, 1.12)	1.03 (0.95, 1.12)	0.97 (0.91, 1.03)
Years post‐cancer diagnosis	0.99 (0.97, 1.01)	0.99 (0.96, 1.02)	0.99 (0.96, 1.01)	0.99 (0.97, 1.01)
Age, years				
66–74	REF	REF	REF	REF
75–84	**1.06 (1.03, 1.10)**	**1.07 (1.01, 1.14)**	**1.12 (1.06, 1.18)**	1.06 (1.02, 1.10)
≥85	**1.07 (1.04, 1.11)**	**1.12 (1.06, 1.20)**	**1.25 (1.19, 1.31)**	1.07 (1.03, 1.11)
Cohort				
1991–1994	REF	REF	REF	REF
1995–1998	**0.88 (0.79, 0.97)**	0.87 (0.70, 1.09)	0.92 (0.81, 1.06)	0.92 (0.77, 1.09)
1999–2002	0.95 (0.82, 1.09)	0.89 (0.67, 1.18)	0.90 (0.73, 1.11)	1.05 (0.86, 1.28)
2003–2006	0.93 (0.77, 1.14)	0.90 (0.62, 1.31)	0.88 (0.66, 1.18)	1.12 (0.87, 1.44)
2007–2011	1.01 (0.78, 1.29)	0.97 (0.60, 1.55)	0.91 (0.62, 1.33)	1.18 (0.86, 1.63)
Gender				
Male	REF	REF	REF	REF
Female	**1.35 (1.25, 1.45)**	**1.51 (1.32, 1.72)**	1.34 (1.19, 1.51)	1.39 (1.28, 1.51)
Race‐ethnicity				
Non‐Hispanic White	REF	REF	REF	REF
Non‐Hispanic Black	**1.30 (1.21, 1.40)**	1.05 (0.94, 1.17)	**1.69 (1.57, 1.82)**	**0.84 (0.80, 0.90)**
Non‐Hispanic Other	**0.38 (0.35, 0.41)**	**0.52 (0.39, 0.71)**	**0.52 (0.39, 0.70)**	**0.45 (0.33, 0.60)**
Hispanic	**0.77 (0.73, 0.82)**	**0.75 (0.65, 0.87)**	0.81 (0.61, 1.06)	**0.73 (0.61, 0.88)**
Cancer diagnosis				
Prostate	REF	REF	REF	REF
Breast	**1.15 (1.05, 1.25)**	1.01 (0.87, 1.18)	1.13 (0.99, 1.29)	1.07 (0.97, 1.18)
Colorectal	**1.14 (1.06, 1.23)**	1.09 (0.96, 1.25)	1.06 (0.95, 1.19)	**1.10 (1.01, 1.19)**
Lung	**1.72 (1.56, 1.89)**	**1.48 (1.26, 1.74)**	**1.44 (1.24, 1.67)**	**1.58 (1.43, 1.74)**
Original reason for entitlement				
Age related	REF	REF	REF	REF
Non‐age related	**2.63 (2.51, 2.76)**	**2.45 (2.24, 2.67)**	**2.16 (2.00, 2.32)**	**2.48 (2.36, 2.62)**
Urban‐rural status				
Metropolis	REF	REF	REF	REF
Urban‐rural	**1.26 (1.19, 1.32)**	1.12 (0.95, 1.31)	0.97 (0.91, 1.03)	**1.34 (1.28, 1.40)**
Medicaid eligibility	**1.85 (1.78, 1.93)**	**1.80 (1.67, 1.93)**	**2.00 (1.89, 2.12)**	**1.79 (1.72, 1.88)**
Charlson's comorbidity ≥1	**1.21 (1.18, 1.23)**	**1.33 (1.26, 1.40)**	**1.27 (1.22, 1.32)**	**1.22 (1.18, 1.25)**
Depressive disorder	**1.20 (1.17, 1.24)**	**1.19 (1.12, 1.26)**	**1.19 (1.13, 1.24)**	**1.14 (1.10, 1.18)**
Anxiety disorder	**1.11 (1.08, 1.15)**	**1.18 (1.12, 1.25)**	**1.12 (1.07, 1.17)**	**1.22 (1.18, 1.26)**
Alcohol use disorder	**1.11 (1.04, 1.19)**	0.94 (0.81, 1.10)	0.98 (0.86, 1.11)	0.97 (0.87, 1.07)
Drug use disorder	**1.49 (1.39, 1.59)**	**1.69 (1.47, 1.96)**	**1.38 (1.24, 1.54)**	**1.56 (1.43, 1.70)**
Opioid naïve	**0.25 (0.25, 0.26)**	**0.17 (0.16, 0.18)**	**0.26 (0.25, 0.27)**	**0.30 (0.29, 0.31)**

Note

Bolded values indicate statistical significance at level of *p* < 0.05.

**FIGURE 2:**
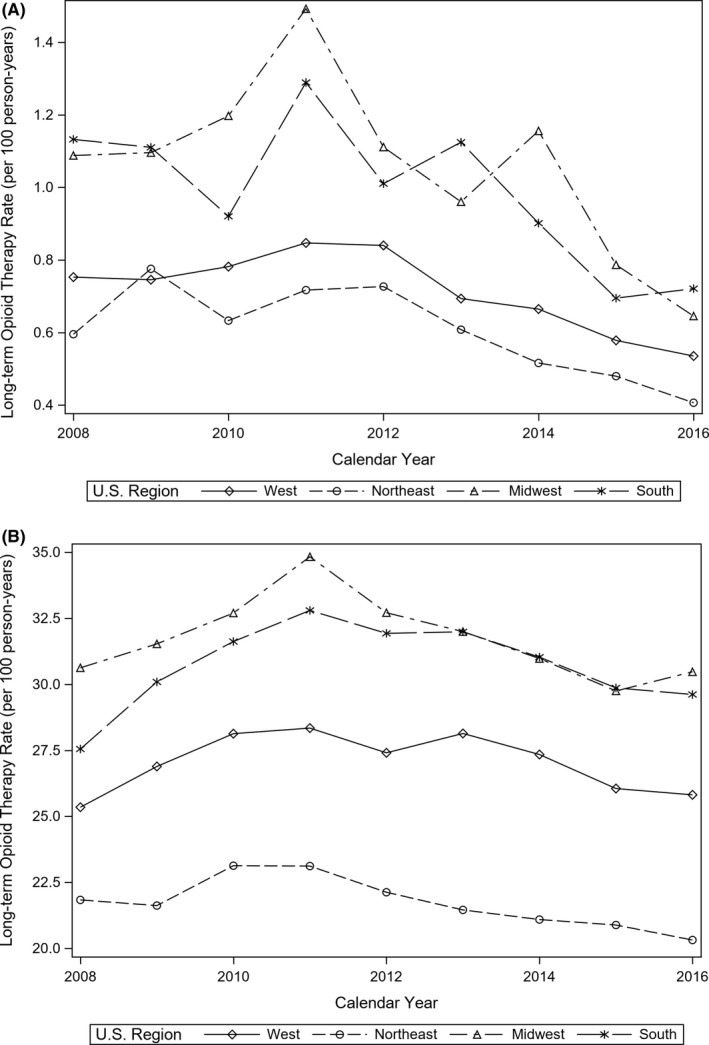
(A) Rate of receipt of long‐term opioid therapy by opioid‐naïve cancer survivors stratified by U.S. census region. (B) Rate of receipt of long‐term opioid therapy by opioid non‐naïve cancer survivors stratified by U.S. census region

The time trend in the rate of long‐term opioid therapy was found to vary significantly by opioid‐naïve status after adjusting for patient demographics, cancer diagnosis, and comorbid conditions (*p* < 0.0001, not shown); therefore, we stratified our models by U.S. census region and opioid‐naïve status. Figure 2 demonstrates the annual rate of long‐term opioid therapy in opioid naïve (Panel A) and opioid non‐naïve (Panel B). The annual time trend in the receipt of long‐term opioid therapy stratified by opioid‐naïve status adjusted for patient demographics and clinical history is presented in Table 3 (for full results, see Tables S3 and S4). After 2013, statistically significant declines were observed in 2014 (aOR = 0.78, 95% CI: 0.64, 0.94), 2015 (aOR = 0.58, 95% CI: 0.47, 0.71), and 2016 (aOR = 0.57, 95% CI: 0.46, 0.71) among opioid‐naïve cancer survivors residing in the south. Similarly, statistically significant declines in long‐term opioid therapy among opioid‐naïve cancer survivors were observed in the Midwest in 2016 (aOR = 0.63, 95% CI: 0.48, 0.82) and in the northeast in 2016 (aOR = 0.71, 95% CI: 0.53, 0.94). Among opioid non‐naïve cancer survivors, statistically significant declines were observed in the west in 2015 (aOR = 0.91, 95% CI: 0.86, 0.95) and 2016 (aOR = 0.87, 95% CI: 0.82, 0.92), in the northeast in 2016 (aOR = 0.87, 95% CI: 0.78, 0.97), in the Midwest in 2015 (aOR = 0.92, 95% CI: 0.86, 1.00), and in the south in 2015 (aOR = 0.90, 95% CI: 0.85, 0.96) and 2016 (aOR = 0.88, 95% CI: 0.82, 0.95).

**TABLE 3:** Adjusted odd ratios (aOR) and 95% confidence intervals of receipt of long‐term opioid therapy stratified by U.S. region and prior opioid use

Calendar year	West	Northeast	Midwest	South
	aOR (95% CI)	aOR (95% CI)	aOR (95% CI)	aOR (95% CI)
Opioid‐naïve subgroup				
2008	0.90 (0.69, 1.16)	0.80 (0.51, 1.25)	1.13 (0.80, 1.60)	1.18 (0.84, 1.66)
2009	0.93 (0.74, 1.16)	1.09 (0.76, 1.57)	1.13 (0.84, 1.54)	1.11 (0.83, 1.48)
2010	1.01 (0.83, 1.23)	0.92 (0.67, 1.27)	1.26 (0.96, 1.65)	0.88 (0.68, 1.15)
2011	1.12 (0.94, 1.34)	1.08 (0.82, 1.42)	**1.61 (1.27, 2.04)**	1.22 (0.98, 1.52)
2012	1.16 (0.98, 1.36)	1.13 (0.88, 1.45)	1.19 (0.93, 1.50)	0.94 (0.76, 1.15)
2013	REF	REF	REF	REF
2014	1.02 (0.88, 1.20)	0.88 (0.69, 1.12)	1.18 (0.95, 1.47)	**0.78 (0.64, 0.94)**
2015	0.92 (0.78, 1.09)	0.83 (0.64, 1.07)	0.80 (0.62, 1.02)	**0.58 (0.47, 0.71)**
2016	0.85 (0.71, 1.02)	**0.71 (0.53, 0.94)**	**0.63 (0.48, 0.82)**	**0.57 (0.46, 0.71)**
Opioid non‐naïve subgroup				
2008	**0.87 (0.79, 0.97)**	1.03 (0.86, 1.25)	0.90 (0.77, 1.06)	**0.86 (0.75, 0.98)**
2009	0.95 (0.87, 1.03)	0.99 (0.85, 1.16)	0.96 (0.84, 1.10)	0.95 (0.86, 1.06)
2010	1.00 (0.94, 1.07)	1.08 (0.95, 1.23)	1.02 (0.92, 1.14)	0.98 (0.90, 1.08)
2011	1.01 (0.96, 1.07)	1.06 (0.96, 1.17)	**1.12 (1.03, 1.22)**	1.04 (0.97, 1.11)
2012	0.96 (0.92, 1.00)	0.99 (0.92, 1.06)	1.03 (0.97, 1.10)	1.00 (0.95, 1.05)
2013	REF	REF	REF	REF
2014	0.98 (0.95, 1.02)	0.95 (0.89, 1.02)	0.97 (0.91, 1.03)	0.97 (0.92, 1.01)
2015	**0.91 (0.86, 0.95)**	0.94 (0.86, 1.02)	**0.92 (0.86, 1.00)**	**0.90 (0.85, 0.96)**
2016	**0.87 (0.82, 0.92)**	**0.87 (0.78, 0.97)**	0.94 (0.85, 1.03)	**0.88 (0.82, 0.95)**

Notes

Models also adjusted for years post‐cancer diagnosis, age, diagnosis cohort, gender, race and ethnicity, cancer diagnosis, original reason for entitlement, urban‐rural status, medicaid eligibility, Charlson's comorbidity ≥1, depressive disorder, anxiety disorder, alcohol use disorder, drug use disorder. Bolded values indicate statistical significance at level *p* < 0.05. For full results, see Tables S3 and S4.

A sensitivity analysis in which observations with less than a full person‐year were removed was consistent with our results, except we did not observe a significant reduction in long‐term opioid use in the Midwest in 2015 among opioid non‐naïve persons (Table S5). We also performed a sensitivity analysis examining the temporal trends within region including only colorectal and lung cancer survivors and found strong declines among opioid‐naïve persons in the south but no significant differences in the other regions among opioid‐naïve and non‐naïve individuals (Table S6). A separate sensitivity analysis exploring temporal trends in receipt of long‐term opioid therapy within each cancer diagnosis group revealed no significant declines after 2013 in all cancer diagnosis groups (Table S7).

## 4 DISCUSSION

We observed that the time trends in the receipt of long‐term opioid therapy among older cancer survivors significantly varied by U.S. region and prior opioid use. Overall, the prevalence of long‐term opioid therapy was highest in the south and lowest in the northeast. After stratifying by previous opioid use, we observed statistically significant and sustained declines in the receipt of long‐term opioid therapy for opioid‐naïve persons residing in the south and among opioid non‐naïve persons in the south and west after 2013. This study builds upon the literature concerning opioid prescribing in older cancer survivors by identifying that time and place are influential contextual factors for receipt of long‐term opioid therapy among older persons who lived 5 or more years after a cancer diagnosis.

In general, previous studies have indicated that opioid prescribing and long‐term opioid therapy rates are lowest in the northeast but highest in the south and have declined substantially after 2010, particularly in the south.^12^, ^13^, ^14^, ^15^, ^16^, ^21^, ^22^, ^23^ Our study cohort was comprised of persons who were diagnosed with cancer in a SEER state or region. SEER capture areas, however, cover approximately 35% of US residents with selected states in different regions.^24^ The long‐term opioid therapy rates among older cancer survivors presented in this study may underestimate actual regional prevalence rates. Most of our cancer survivor cohort in the west (California, Washington), northeast (New Jersey, Connecticut), Midwest (Michigan, Iowa), and south (Georgia, Kentucky, Louisiana) resided in states with lower rates of long‐term opioid therapy compared with some regional non‐SEER neighboring states, although some SEER states historically had high long‐term use rates. There were fewer observations in our study from non‐SEER states with observed high long‐term opioid therapy rates. Despite these differences, our results on the geographical patterning of long‐term opioid therapy rates among older cancer survivors are consistent with the findings of long‐term opioid therapy in Medicare beneficiaries.^23^

Gender differences in the utilization of prescription opioids have been previously observed in older adults, with women more likely to receive long‐term opioid therapy and men more likely to receive high‐dose therapy, but this is debated.^22^, ^23^, ^25^, ^26^ Temporal trends in long‐term opioid use have found larger absolute and relative reductions in the rates of long‐term opioid use in women as compared to men.^23^ Our study is consistent with Shah et al. (2019), which found that female cancer survivors are more likely to receive long‐term opioid therapy than men.^4^ We performed a sensitivity analysis examining the trends of long‐term opioid use within regions only in persons diagnosed with colorectal or lung cancer to reduce the influence of breast and prostate cancer survivors on our results. We found strong declines among opioid‐naïve persons in the south but did not observe declines in other regions regardless of previous opioid use. One reason for the difference in the results between the main findings and the sensitivity analysis could be that colorectal and lung cancer survivors were more likely to be diagnosed with metastatic tumors that extended regionally or distantly and therefore experience more adverse consequences related to treatment and requiring more prescription opioid use in survivorship.

There are several possible explanations for the reduction in long‐term opioid therapy rates observed in this study. Declines in the rate of long‐term opioid therapy may be associated with the rescheduling of hydrocodone combination products (HCP) from schedule III to the more restrictive schedule II by the Drug Enforcement Administration in October 2014.^20^, ^27^, ^28^, ^29^, ^30^ However, in an unadjusted analysis, we noted that declines in the rate of long‐term opioid therapy preceded the enforcement of hydrocodone reclassification. We also observed regional variation in the declines of long‐term opioid use after HCP rescheduling, despite HCP rescheduling being a broad federal policy initiative. Statewide variation in the relative reductions of HCP prescribing and receipt of long‐term opioid therapy after the enforcement of HCP rescheduling has been previously observed,^23^, ^27^ but regional differences in receipt of long‐term opioid therapy could also be associated with state prescription drug monitoring program policy and changes to hospital and insurance organizational guidelines restricting opioid prescribing.

During the study period, state legislatures, governors, and medical boards aimed to reduce opioid prescribing by implementing policies, rules, and guidelines that attempted to change prescriber behavior. Some commonly enacted state policies regulated pain clinics, limited the initial amount or days supplied of opioids, and mandated providers to check the prescription drug monitoring program (PDMP) before prescribing opioids.^31^, ^32^, ^33^, ^34^, ^35^, ^36^, ^37^, ^38^ However, regional and temporal differences in the implementation of legislation and regulations have been noted.^34^, ^35^, ^36^, ^37^, ^38^ For example, southern states were early adopters that required providers to enroll into a PDMP and review patient's opioid prescriptions—at least in some circumstances—before prescribing an opioid. Moreover, many southern states instituted strict regulations on pain clinics that specified clinic ownership, registration with the state, and best clinical practices. Some Midwestern states limited the daily amount of opioids prescribed but did not, in general, require PDMP review. Many northeastern states adopted policies mandating PDMP enrollment and use, and imposed limitations on the days supplied of prescription opioids for initial prescriptions. Similarly, legislation requiring PDMP enrollment and restricting the daily amount of opioids prescribed or dispensed was common in Western states.

We did not observe that states with most of the person‐time observed in our study were more likely to require providers use PDMP programs than states with smaller percentage of cancer survivors. Through 2015, only 10 states clustered in the northeast and Ohio River Valley and 3 states west of the Mississippi River had legislation mandating all providers to check the PDMP before an initial opioid prescription (Nevada, New Mexico, Oklahoma, Kentucky, Tennessee, West Virginia, Ohio, Pennsylvania, New Jersey, New York, Connecticut, Rhode Island, Massachusetts).^34^ Further studies should be conducted to examine how state policies interact with federal policy to reduce opioid prescribing.

In 2016, the CDC and American Society of Clinical Oncology (ASCO) released guidelines on opioid prescribing for chronic pain and recommended the use of non‐opioid analgesics and non‐pharmacological treatment of chronic pain.^39^, ^40^ The dissemination of these guidelines, however, cannot explain declines in receipt of long‐term opioid therapy that were observed to begin around 2014 in some regions but could explain some of the decline noted in 2016 in the northeast and Midwest. Our study did not have a long enough follow‐up time to isolate the effects of these guidelines on the rates of long‐term opioid therapy. Furthermore, many insurers, hospital systems, and pharmacies implemented organizational guidelines to reduce opioid prescribing and dispensing. We are unable to examine how these organizational changes affected trends in long‐term opioid therapy. Lastly, provider attitudes toward prescription opioids may have changed over time because of the reports on the increase in morbidity and mortality associated with prescription opioid use. Early reports suggested that physicians expressed relatively little concern about the addiction and dependence potential of prescription opioids, but recent surveys have shown greater concerns over opioid misuse.^41^, ^42^, ^43^

This study has several limitations. First, we used administrative claims data from Medicare from persons who were diagnosed with breast, colorectal, lung, or prostate cancer in a SEER region, lived at least 5 years post‐diagnosis, continuously enrolled in Part A, B, and D, and did not receive cancer treatment or hospice care. Our results are not generalizable to other cancer survivor populations and individuals who were enrolled in an HMO, or were not diagnosed in a SEER region. Second, opioids that were not prescribed to a person in our cohort or opioids prescribed but not dispensed through Part D could not be counted toward total days of having an opioid prescription. Third, our analysis assumes the prescription for opioids was taken as directed. Fourth, our study using administrative claims data is not able to link prescription opioid utilization to patient‐reported pain severity or personal beliefs on prescription opioids. Fifth, our study did not have a large enough sample size of persons alive ≥5 years post‐cancer diagnosis to conduct a state policy analysis. Sixth, we were unable to examine how current disease—5 or more years after diagnosis—was associated with receipt of long‐term opioid therapy. We attempted to address this limitation by excluding individuals if they were diagnosed with a second primary cancer and by requiring that persons be not receiving chemotherapy or radiation. Last, this study did not include information about opioid prescribers. The differences in receipt of long‐term opioid therapy may be related to distribution of providers caring for cancer survivors or due to intra‐specialty temporal trends in opioid prescribing. Future studies are needed to examine how opioid prescribing by providers changes over time and across regions.

This study has several strengths. This study utilizes information from multiple and geographically diverse regions or state‐based registries with high capture rates for cases linked with Part A, B, and D Medicare claims. This allows for detailed follow‐up using reliable information. Moreover, we could follow individuals if they moved to another state or region, which allowed us to assess variation in long‐term opioid therapy by a person's residence over time. Lastly, we were able to censor individuals at the time of death or receipt of hospice care or assess whether they received treatment for cancer in the year prior to follow‐up.

In conclusion, we found evidence that the rates of long‐term opioid therapy varied by time, geographical region, and previous opioid use for older cancer survivors. Receipt of long‐term opioid therapy was the highest in the south and lowest in the northeast. Variation in a clinical practice suggests the need for more research and interventions to improve efficiency, process, cost, and quality of care.^44^ Research should explore what factors explain the geographical variation in prescribing, and what policy and public health interventions are needed to reduce high rates of long‐term opioid therapy for the growing number of older long‐term cancer survivors.

## CONFLICT OF INTEREST

The authors declare no potential conflicts of interest.

## Supporting information

Supplementary Material

## Data Availability

The data that support the findings of this study are available from National Cancer Institute, the Centers for Medicare and Medicaid Services, and SEER. Restrictions apply to the availability of these data, which were used under license for this study.
