# Supplementary material for: Regional and temporal variation in receipt of long‐term opioid therapy among older breast, colorectal, lung, and prostate cancer survivors in the United States
Source: Cancer Med. 2021 Jan 9;10(5):1550–61. doi: 10.1002/cam4.3709 (PMC7940244; doi:10.1002/cam4.3709)
Supplement: Supplementary file 1 — Supplementary Material [file CAM4-10-1550-s001.docx]

**Supplementary Figure 1**

Persons diagnosed from 1991-2011, no autopsy or death certificate, no missing date information, and no secondary cancer diagnosis and lived at least 5 years (n=1,033,292)

Had at least 12 months of Part A, B, and D enrollment before a calendar year (n=413,548)

No cancer treatment in 12 months before a calendar year (n=386,023)

Lived or did not receive hospice care prior to April 01 (n=380,583)

Aged ≥66 years of age during a calendar year (n=360,531)

Continuous Part A, B, D enrollment for 0.25 persons years if deceased or hospice or 1 person-year if uncensored (n=344,744)

Persons who with complete information on state of residence and urban-rural status (n=344,443)

**Supplementary Table 1.**

|  | Chemotherapy | Radiation |
| --- | --- | --- |
| ICD-9 Diagnosis Codes | V581, V662, V672 | V580, V661, V671 |
| ICD-10 Diagnosis Codes | Z5111, Z5112 | Z510 |
| ICD-9 Procedure Codes | 9925 | 9221-9229 |
| ICD-10 Procedure Codes** | 3E03305, 3E04305, XW03351, XW04351 | **See Radiation ICD-10 Procedure Codes Below** |
| Revenue Center Codes |  | 0330, 0333 |
| CPT/HCPCS Codes | 964xx, 96400-96549, Q0083-Q0085, 51720, J9000-J9999 | 77401-77499, 77520, 77523, 77750-77799, G0265, G0261 |

ICD-10-CM codes based on crosswalking ICD-9-CM to ICD-10-CM codes and guidance provided by NCI: SEER Training Modules (<https://healthcaredelivery.cancer.gov/seermedicare/considerations/procedure_codes.html>, <https://training.seer.cancer.gov/icd10cm/appendix-a/>).

**Primary hospitalization with diagnosis of cancer identified with the following codes**

140 ,141 ,142 ,143 ,144 ,145 ,146 ,147 ,148, 149 ,150 ,151 ,152 ,153 ,154 ,155 ,156 ,157 ,158 ,159 ,160 ,161 ,162 ,163 ,164 ,165 ,170, 171 ,172 ,174 ,175 ,176 ,179 ,180 ,181 ,182 ,183 ,184 ,185 ,186 ,187 ,188 ,189 ,190 ,191, 192 ,193 ,194 ,195 ,200 ,201 ,202 ,203 ,204 ,205 ,206 ,207 ,208 ,196 ,197 ,198 ,199 ,2386 ,C00 ,C01 ,C02 ,C03 ,C04 ,C05 ,C06 ,C07 ,C08 ,C09 ,C10 ,C11 ,C12 ,C13 ,C14 ,C15 ,C16 ,C17 ,C18 ,C19 ,C20 ,C21 ,C22 ,C23 ,C24 ,C25 ,C26 ,C30 ,C31 ,C32 ,C33 ,C34 ,C37 ,C38 ,C39 ,C40 ,C41 ,C43 ,C45 ,C46 ,C47 ,C48 ,C49 ,C50 ,C51 ,C52 ,C53 ,C54 ,C55 ,C56 ,C57 ,C58 ,C60 ,C61 ,C62 ,C63 ,C64 ,C65 ,C66 ,C67 ,C68 ,C69 ,C70 ,C71 ,C72 ,C73 ,C74 ,C75 ,C76 ,C77, C78 ,C79 ,C80 C81 ,C82 ,C83 ,C84, C85 ,C88 ,C90, C91 ,C92, C93 ,C94 ,C95 ,C96 ,C97

**Radiation ICD-10-Procedure Codes**

D0000ZZ, D0010ZZ, D0060ZZ, D0070ZZ, D7000ZZ, D7010ZZ, D7020ZZ, D7030ZZ, D7040ZZ, D7050ZZ, D7060ZZ, D7070ZZ, D7080ZZ, D8000ZZ, D9000ZZ, D9010ZZ, D9030ZZ, D9040ZZ, D9050ZZ, D9060ZZ, D9070ZZ, D9080ZZ, D9090ZZ, D90B0ZZ, D90D0ZZ, D90F0ZZ, DB000ZZ, DB010ZZ, DB020ZZ, DB050ZZ, DB060ZZ, DB070ZZ, DB080ZZ, DD000ZZ, DD010ZZ, DD020ZZ, DD030ZZ, DD040ZZ, DD050ZZ, DD070ZZ, DF000ZZ, DF010ZZ, DF020ZZ, DF030ZZ, DG000ZZ, DG010ZZ, DG020ZZ, DG040ZZ, DG050ZZ, DH020ZZ, DH030ZZ, DH040ZZ, DH060ZZ, DH070ZZ, DH080ZZ, DH090ZZ, DH0B0ZZ, DM000ZZ, DM010ZZ, DP000ZZ, DP020ZZ, DP030ZZ, DP040ZZ, DP050ZZ, DP060ZZ, DP070ZZ, DP080ZZ, DP090ZZ, DP0B0ZZ, DP0C0ZZ, DT000ZZ, DT010ZZ, DT020ZZ, DT030ZZ, DU000ZZ, DU010ZZ, DU020ZZ, DV000ZZ, DV010ZZ, DW010ZZ, DW020ZZ, DW030ZZ, DW040ZZ, DW050ZZ, DW060ZZ, D0000ZZ, D0001ZZ, D0002ZZ, D0010ZZ, D0011ZZ, D0012ZZ, D0060ZZ, D0061ZZ, D0062ZZ, D0070ZZ, D0071ZZ, D0072ZZ, D7000ZZ, D7001ZZ, D7002ZZ, D7010ZZ, D7011ZZ, D7012ZZ, D7020ZZ, D7021ZZ, D7022ZZ, D7030ZZ, D7031ZZ, D7032ZZ, D7040ZZ, D7041ZZ, D7042ZZ, D7050ZZ, D7051ZZ, D7052ZZ, D7060ZZ, D7061ZZ, D7062ZZ, D7070ZZ, D7071ZZ, D7072ZZ, D7080ZZ, D7081ZZ, D7082ZZ, D8000ZZ, D8001ZZ, D8002ZZ, D9000ZZ, D9001ZZ, D9002ZZ, D9010ZZ, D9011ZZ, D9012ZZ, D9030ZZ, D9031ZZ, D9032ZZ, D9040ZZ, D9041ZZ, D9042ZZ, D9050ZZ, D9051ZZ, D9052ZZ, D9060ZZ, D9061ZZ, D9062ZZ, D9070ZZ, D9071ZZ, D9072ZZ, D9080ZZ, D9081ZZ, D9082ZZ, D9090ZZ, D9091ZZ, D9092ZZ, D90B0ZZ, D90B1ZZ, D90B2ZZ, D90D0ZZ, D90D1ZZ, D90D2ZZ, D90F0ZZ, D90F1ZZ, D90F2ZZ, DB000ZZ, DB001ZZ, DB002ZZ, DB010ZZ, DB011ZZ, DB012ZZ, DB020ZZ, DB021ZZ, DB022ZZ, DB050ZZ, DB051ZZ, DB052ZZ, DB060ZZ, DB061ZZ, DB062ZZ, DB070ZZ, DB071ZZ, DB072ZZ, DB080ZZ, DB081ZZ, DB082ZZ, DD000ZZ, DD001ZZ, DD002ZZ, DD010ZZ, DD011ZZ, DD012ZZ, DD020ZZ, DD021ZZ, DD022ZZ, DD030ZZ, DD031ZZ, DD032ZZ, DD040ZZ, DD041ZZ, DD042ZZ, DD050ZZ, DD051ZZ, DD052ZZ, DD070ZZ, DD071ZZ, DD072ZZ, DF000ZZ, DF001ZZ, DF002ZZ, DF010ZZ, DF011ZZ, DF012ZZ, DF020ZZ, DF021ZZ, DF022ZZ, DF030ZZ, DF031ZZ, DF032ZZ, DG000ZZ, DG001ZZ, DG002ZZ, DG010ZZ, DG011ZZ, DG012ZZ, DG020ZZ, DG021ZZ, DG022ZZ, DG040ZZ, DG041ZZ, DG042ZZ, DG050ZZ, DG051ZZ, DG052ZZ, DH020ZZ, DH021ZZ, DH022ZZ, DH030ZZ, DH031ZZ, DH032ZZ, DH040ZZ, DH041ZZ, DH042ZZ, DH060ZZ, DH061ZZ, DH062ZZ, DH070ZZ, DH071ZZ, DH072ZZ, DH080ZZ, DH081ZZ, DH082ZZ, DH090ZZ, DH091ZZ, DH092ZZ, DH0B0ZZ, DH0B1ZZ, DH0B2ZZ, DM000ZZ, DM001ZZ, DM002ZZ, DM010ZZ, DM011ZZ, DM012ZZ, DP000ZZ, DP001ZZ, DP002ZZ, DP020ZZ, DP021ZZ, DP022ZZ, DP030ZZ, DP031ZZ, DP032ZZ, DP040ZZ, DP041ZZ, DP042ZZ, DP050ZZ, DP051ZZ, DP052ZZ, DP060ZZ, DP061ZZ, DP062ZZ, DP070ZZ, DP071ZZ, DP072ZZ, DP080ZZ, DP081ZZ, DP082ZZ, DP090ZZ, DP091ZZ, DP092ZZ, DP0B0ZZ, DP0B1ZZ, DP0B2ZZ, DP0C0ZZ, DP0C1ZZ, DP0C2ZZ, DT000ZZ, DT001ZZ, DT002ZZ, DT010ZZ, DT011ZZ, DT012ZZ, DT020ZZ, DT021ZZ, DT022ZZ, DT030ZZ, DT031ZZ, DT032ZZ, DU000ZZ, DU001ZZ, DU002ZZ, DU010ZZ, DU011ZZ, DU012ZZ, DU020ZZ, DU021ZZ, DU022ZZ, DV000ZZ, DV001ZZ, DV002ZZ, DV010ZZ, DV011ZZ, DV012ZZ, DW010ZZ, DW011ZZ, DW012ZZ, DW020ZZ, DW021ZZ, DW022ZZ, DW030ZZ, DW031ZZ, DW032ZZ, DW040ZZ, DW041ZZ, DW042ZZ, DW050ZZ, DW051ZZ, DW052ZZ, DW060ZZ, DW061ZZ, DW062ZZ, D0003ZZ, D0013ZZ, D0063ZZ, D0073ZZ, D7003ZZ, D7013ZZ, D7023ZZ, D7033ZZ, D7043ZZ, D7053ZZ, D7063ZZ, D7073ZZ, D7083ZZ, D8003ZZ, D9003ZZ, D9013ZZ, D9033ZZ, D9043ZZ, D9053ZZ, D9063ZZ, D9073ZZ, D9083ZZ, D9093ZZ, D90B3ZZ, D90D3ZZ, D90F3ZZ, DB003ZZ, DB013ZZ, DB023ZZ, DB053ZZ, DB063ZZ, DB073ZZ, DB083ZZ, DD003ZZ, DD013ZZ, DD023ZZ, DD033ZZ, DD043ZZ, DD053ZZ, DD073ZZ, DF003ZZ, DF013ZZ, DF023ZZ, DF033ZZ, DG003ZZ, DG013ZZ, DG023ZZ, DG043ZZ, DG053ZZ, DH023ZZ, DH033ZZ, DH043ZZ, DH063ZZ, DH073ZZ, DH083ZZ, DH093ZZ, DH0B3ZZ, DM003ZZ, DM013ZZ, DP003ZZ, DP023ZZ, DP033ZZ, DP043ZZ, DP053ZZ, DP063ZZ, DP073ZZ, DP083ZZ, DP093ZZ, DP0B3ZZ, DP0C3ZZ, DT003ZZ, DT013ZZ, DT023ZZ, DT033ZZ, DU003ZZ, DU013ZZ, DU023ZZ, DV003ZZ, DV013ZZ, DW013ZZ, DW023ZZ, DW033ZZ, DW043ZZ, DW053ZZ, DW063ZZ, D0004ZZ, D0005ZZ, D0014ZZ, D0015ZZ, D0064ZZ, D0065ZZ, D0074ZZ, D0075ZZ, D7004ZZ, D7005ZZ, D7014ZZ, D7015ZZ, D7024ZZ, D7025ZZ, D7034ZZ, D7035ZZ, D7044ZZ, D7045ZZ, D7054ZZ, D7055ZZ, D7064ZZ, D7065ZZ, D7074ZZ, D7075ZZ, D7084ZZ, D7085ZZ, D8004ZZ, D8005ZZ, D9004ZZ, D9005ZZ, D9014ZZ, D9015ZZ, D9034ZZ, D9035ZZ, D9044ZZ, D9045ZZ, D9054ZZ, D9055ZZ, D9064ZZ, D9065ZZ, D9074ZZ, D9075ZZ, D9084ZZ, D9085ZZ, D9094ZZ, D9095ZZ, D90B4ZZ, D90B5ZZ, D90D4ZZ, D90D5ZZ, D90F4ZZ, D90F5ZZ, DB004ZZ, DB005ZZ, DB014ZZ, DB015ZZ, DB024ZZ, DB025ZZ, DB054ZZ, DB055ZZ, DB064ZZ, DB065ZZ, DB074ZZ, DB075ZZ, DB084ZZ, DB085ZZ, DD004ZZ, DD005ZZ, DD014ZZ, DD015ZZ, DD024ZZ, DD025ZZ, DD034ZZ, DD035ZZ, DD044ZZ, DD045ZZ, DD054ZZ, DD055ZZ, DD074ZZ, DD075ZZ, DF004ZZ, DF005ZZ, DF014ZZ, DF015ZZ, DF024ZZ, DF025ZZ, DF034ZZ, DF035ZZ, DG005ZZ, DG015ZZ, DG025ZZ, DG045ZZ, DG055ZZ, DH024ZZ, DH025ZZ, DH034ZZ, DH035ZZ, DH044ZZ, DH045ZZ, DH064ZZ, DH065ZZ, DH074ZZ, DH075ZZ, DH084ZZ, DH085ZZ, DH094ZZ, DH095ZZ, DH0B4ZZ, DH0B5ZZ, DM004ZZ, DM005ZZ, DM014ZZ, DM015ZZ, DP004ZZ, DP005ZZ, DP024ZZ, DP025ZZ, DP034ZZ, DP035ZZ, DP044ZZ, DP045ZZ, DP054ZZ, DP055ZZ, DP064ZZ, DP065ZZ, DP074ZZ, DP075ZZ, DP084ZZ, DP085ZZ, DP094ZZ, DP095ZZ, DP0B4ZZ, DP0B5ZZ, DP0C4ZZ, DP0C5ZZ, DT004ZZ, DT005ZZ, DT014ZZ, DT015ZZ, DT024ZZ, DT025ZZ, DT034ZZ, DT035ZZ, DU004ZZ, DU005ZZ, DU014ZZ, DU015ZZ, DU024ZZ, DU025ZZ, DV004ZZ, DV005ZZ, DV014ZZ, DV015ZZ, DW014ZZ, DW015ZZ, DW024ZZ, DW025ZZ, DW034ZZ, DW035ZZ, DW044ZZ, DW045ZZ, DW054ZZ, DW055ZZ, DW064ZZ, DW065ZZ, 08H031Z, 08H0X1Z, 08H131Z, 08H1X1Z, 0BH001Z, 0BH031Z, 0BH041Z, 0BH071Z, 0BH081Z, 0BHK01Z, 0BHK31Z, 0BHK41Z, 0BHK71Z, 0BHK81Z, 0BHL01Z, 0BHL31Z, 0BHL41Z, 0BHL71Z, 0BHL81Z, 0CH701Z, 0CH731Z, 0CH7X1Z, 0DH501Z, 0DH531Z, 0DH541Z, 0DH571Z, 0DH581Z, 0DHP01Z, 0DHP31Z, 0DHP41Z, 0DHP71Z, 0DHP81Z, 0FHB01Z, 0FHB31Z, 0FHB41Z, 0FHB71Z, 0FHB81Z, 0FHD01Z, 0FHD31Z, 0FHD41Z, 0FHD71Z, 0FHD81Z, 0HHT01Z, 0HHT31Z, 0HHT71Z, 0HHT81Z, 0HHTX1Z, 0HHU01Z, 0HHU31Z, 0HHU71Z, 0HHU81Z, 0HHUX1Z, 0HHV01Z, 0HHV31Z, 0HHV71Z, 0HHV81Z, 0HHVX1Z, 0HHW01Z, 0HHW31Z, 0HHW71Z, 0HHW81Z, 0HHWX1Z, 0HHX01Z, 0HHX31Z, 0HHX71Z, 0HHX81Z, 0HHXX1Z, 0JHS01Z, 0JHS31Z, 0JHT01Z, 0JHT31Z, 0JHV01Z, 0JHV31Z, 0JHW01Z, 0JHW31Z, 0UHC01Z, 0UHC31Z, 0UHC41Z, 0UHC71Z, 0UHC81Z, 0UHG01Z, 0UHG31Z, 0UHG41Z, 0UHG71Z, 0UHG81Z, 0UHGX1Z, 0VH001Z, 0VH031Z, 0VH041Z, 0VH071Z, 0VH081Z, 0WH001Z, 0WH031Z, 0WH041Z, 0WH101Z, 0WH131Z, 0WH141Z, 0WH201Z, 0WH231Z, 0WH241Z, 0WH301Z, 0WH331Z, 0WH341Z, 0WH401Z, 0WH431Z, 0WH441Z, 0WH501Z, 0WH531Z, 0WH541Z, 0WH601Z, 0WH631Z, 0WH641Z, 0WH801Z, 0WH831Z, 0WH841Z, 0WH901Z, 0WH931Z, 0WH941Z, 0WHB01Z, 0WHB31Z, 0WHB41Z, 0WHC01Z, 0WHC31Z, 0WHC41Z, 0WHD01Z, 0WHD31Z, 0WHD41Z, 0WHF01Z, 0WHF31Z, 0WHF41Z, 0WHG01Z, 0WHG31Z, 0WHG41Z, 0WHH01Z, 0WHH31Z, 0WHH41Z, 0WHJ01Z, 0WHJ31Z, 0WHJ41Z, 0WHK01Z, 0WHK31Z, 0WHK41Z, 0WHL01Z, 0WHL31Z, 0WHL41Z, 0WHM01Z, 0WHM31Z, 0WHM41Z, 0WHN01Z, 0WHN31Z, 0WHN41Z, 0WHP01Z, 0WHP31Z, 0WHP41Z, 0WHP71Z, 0WHP81Z, 0WHQ01Z, 0WHQ31Z, 0WHQ41Z, 0WHQ71Z, 0WHQ81Z, 0WHR01Z, 0WHR31Z, 0WHR41Z, 0WHR71Z, 0WHR81Z, 0XH201Z, 0XH231Z, 0XH241Z, 0XH301Z, 0XH331Z, 0XH341Z, 0XH401Z, 0XH431Z, 0XH441Z, 0XH501Z, 0XH531Z, 0XH541Z, 0XH601Z, 0XH631Z, 0XH641Z, 0XH701Z, 0XH731Z, 0XH741Z, 0XH801Z, 0XH831Z, 0XH841Z, 0XH901Z, 0XH931Z, 0XH941Z, 0XHB01Z, 0XHB31Z, 0XHB41Z, 0XHC01Z, 0XHC31Z, 0XHC41Z, 0XHD01Z, 0XHD31Z, 0XHD41Z, 0XHF01Z, 0XHF31Z, 0XHF41Z, 0XHG01Z, 0XHG31Z, 0XHG41Z, 0XHH01Z, 0XHH31Z, 0XHH41Z, 0XHJ01Z, 0XHJ31Z, 0XHJ41Z, 0XHK01Z, 0XHK31Z, 0XHK41Z, 0YH001Z, 0YH031Z, 0YH041Z, 0YH101Z, 0YH131Z, 0YH141Z, 0YH501Z, 0YH531Z, 0YH541Z, 0YH601Z, 0YH631Z, 0YH641Z, 0YH701Z, 0YH731Z, 0YH741Z, 0YH801Z, 0YH831Z, 0YH841Z, 0YH901Z, 0YH931Z, 0YH941Z, 0YHB01Z, 0YHB31Z, 0YHB41Z, 0YHC01Z, 0YHC31Z, 0YHC41Z, 0YHD01Z, 0YHD31Z, 0YHD41Z, 0YHF01Z, 0YHF31Z, 0YHF41Z, 0YHG01Z, 0YHG31Z, 0YHG41Z, 0YHH01Z, 0YHH31Z, 0YHH41Z, 0YHJ01Z, 0YHJ31Z, 0YHJ41Z, 0YHK01Z, 0YHK31Z, 0YHK41Z, 0YHL01Z, 0YHL31Z, 0YHL41Z, 0YHM01Z, 0YHM31Z, 0YHM41Z, 0YHN01Z, 0YHN31Z, 0YHN41Z, CW70NZZ, CW70YZZ, CW73NZZ, CW73YZZ, CW7GGZZ, CW7GYZZ, CW7N8ZZ, CW7NGZZ, CW7NNZZ, CW7NPZZ, CW7NYZZ, CW7YYZZ, DWY5GDZ, DWY5GFZ, DWY5GGZ, DWY5GHZ, DWY5GYZ, D0Y0FZZ, D0Y1FZZ, D0Y6FZZ, D0Y7FZZ, D7Y0FZZ, D7Y1FZZ, D7Y2FZZ, D7Y3FZZ, D7Y4FZZ, D7Y5FZZ, D7Y6FZZ, D7Y7FZZ, D7Y8FZZ, D8Y0FZZ, D9Y0FZZ, D9Y1FZZ, D9Y4CZZ, D9Y4FZZ, D9Y5FZZ, D9Y6FZZ, D9Y7FZZ, D9Y8FZZ, D9Y9FZZ, D9YBCZZ, D9YBFZZ, D9YCCZZ, D9YCFZZ, D9YDCZZ, D9YDFZZ, DBY0FZZ, DBY1FZZ, DBY2FZZ, DBY5FZZ, DBY6FZZ, DBY7FZZ, DBY8FZZ, DDY0FZZ, DDY1CZZ, DDY1FZZ, DDY2CZZ, DDY2FZZ, DDY3CZZ, DDY3FZZ, DDY4CZZ, DDY4FZZ, DDY5CZZ, DDY5FZZ, DDY7CZZ, DDY7FZZ, DDY8CZZ, DDY8FZZ, DFY0CZZ, DFY0FZZ, DFY1CZZ, DFY1FZZ, DFY2CZZ, DFY2FZZ, DFY3CZZ, DFY3FZZ, DGY0FZZ, DGY1FZZ, DGY2FZZ, DGY4FZZ, DGY5FZZ, DHY2FZZ, DHY3FZZ, DHY4FZZ, DHY5FZZ, DHY6FZZ, DHY7FZZ, DHY8FZZ, DHY9FZZ, DHYBFZZ, DHYCFZZ, DMY0FZZ, DMY1FZZ, DPY0FZZ, DPY2FZZ, DPY3FZZ, DPY4FZZ, DPY5FZZ, DPY6FZZ, DPY7FZZ, DPY8FZZ, DPY9FZZ, DPYBFZZ, DPYCFZZ, DTY0CZZ, DTY0FZZ, DTY1CZZ, DTY1FZZ, DTY2CZZ, DTY2FZZ, DTY3CZZ, DTY3FZZ, DUY0CZZ, DUY0FZZ, DUY1CZZ, DUY1FZZ, DUY2CZZ, DUY2FZZ, DVY0CZZ, DVY0FZZ, DVY1FZZ, DWY1FZZ, DWY2FZZ, DWY3FZZ, DWY4FZZ, DWY5FZZ, DWY6FZZ,

**Supplementary Table 2**

| **Variables** | **2008 (n=74773)** | **2009 (n=86666)** | **2010 (n=97355)** | **2011 (n=111243)** | **2012 (n=128691)** | **2013 (n=152846)** | **2014 (n=190539)** | **2015 (n=209667)** | **2016 (n=226417)** |
| --- | --- | --- | --- | --- | --- | --- | --- | --- | --- |
| **Age mean(std)** | 78.4 (7.5) | 78.4 (7.5) | 78.3 (7.6) | 78.3 (7.6) | 78.2 (7.5) | 78.0 (7.5) | 78.0 (7.5) | 77.9 (7.5) | 77.8 (7.4) |
| **Age median(IQR)** | 78.0 (72.2, 83.8) | 77.8 (72.1, 83.8) | 77.8 (72.0, 83.8) | 77.5 (72.0, 83.8) | 77.4 (72.0, 83.6) | 77.1 (71.9, 83.5) | 77.0 (71.8, 83.4) | 77.0 (71.9, 83.3) | 76.9 (71.9, 83.1) |
| **Age, Categorical** |  |  |  |  |  |  |  |  |  |
| 66-74 years | 24490 (32.9%) | 28890 (33.5%) | 32903 (34.0%) | 38017 (34.4%) | 44080 (34.6%) | 53016 (35.1%) | 67024 (35.6%) | 74271 (35.8%) | 83878 (36.1%) |
| 75-84 years | 28961 (38.9%) | 32678 (37.9%) | 36138 (37.4%) | 40909 (37.1%) | 47029 (36.9%) | 55689 (36.9%) | 69745 (37.0%) | 76578 (37.0%) | 85678 (36.9%) |
| ≥85 years | 24490 (32.9%) | 28890 (33.5%) | 32903 (34.0%) | 38017 (34.4%) | 36227 (28.4%) | 42411 (28.1%) | 51636 (27.4%) | 56395 (27.2%) | 62624 (27.0%) |
| **Years Post Cancer Diagnosis** |  |  |  |  |  |  |  |  |  |
| Mean(std) | 9.0 (3.3) | 9.3 (3.4) | 9.7 (3.6) | 10.0 (3.7) | 10.3 (3.9) | 10.6 (4.1) | 11.0 (4.3) | 11.3 (4.4) | 11.7 (4.6) |
| Median(IQR) | 7.8 (6.3, 11.4) | 8.3 (6.7, 11.6) | 8.8 (6.8, 11.8) | 9.2 (7.0, 11.9) | 9.6 (7.2, 12.2) | 9.9 (7.3, 12.7) | 10.3 (7.5, 13.3) | 10.7 (7.7, 13.9) | 11.0 (7.9, 14.6) |
| **Gender** |  |  |  |  |  |  |  |  |  |
| Female | 40640 (54.5%) | 46743 (54.2%) | 52109 (53.9%) | 59243 (53.7%) | 67317 (52.9%) | 78332 (51.8%) | 95895 (50.9%) | 105338 (50.8%) | 117976 (50.8%) |
| Male | 33868 (45.5%) | 39465 (45.8%) | 44578 (46.1%) | 51127 (46.3%) | 60019 (47.1%) | 72784 (48.2%) | 92510 (49.1%) | 101906 (49.2%) | 114204 (49.2%) |
| **Race and Ethnicity** |  |  |  |  |  |  |  |  |  |
| Hispanic | 4507 (6.0%) | 5506 (6.4%) | 6360 (6.6%) | 7214 (6.5%) | 8081 (6.3%) | 9068 (6.0%) | 10081 (5.4%) | 10655 (5.1%) | 12076 (5.2%) |
| Non-Hispanic Black | 5084 (6.8%) | 5954 (6.9%) | 6422 (6.6%) | 7326 (6.6%) | 8412 (6.6%) | 10175 (6.7%) | 14159 (7.5%) | 15011 (7.2%) | 16249 (7.0%) |
| Non-Hispanic Other | 5197 (7.0%) | 5826 (6.8%) | 6577 (6.8%) | 7603 (6.9%) | 8612 (6.8%) | 9790 (6.5%) | 11373 (6.0%) | 12260 (5.9%) | 14276 (6.1%) |
| Non-Hispanic White | 59720 (80.2%) | 68922 (79.9%) | 77328 (80.0%) | 88227 (79.9%) | 102231 (80.3%) | 122083 (80.8%) | 152792 (81.1%) | 169318 (81.7%) | 189579 (81.7%) |
| **Cancer Diagnosis** |  |  |  |  |  |  |  |  |  |
| Breast Cancer | 29896 (40.1%) | 34724 (40.3%) | 39063 (40.4%) | 44703 (40.5%) | 51232 (40.2%) | 60343 (39.9%) | 75001 (39.8%) | 83172 (40.1%) | 94149 (40.6%) |
| Colorectal Cancer | 14364 (19.3%) | 16214 (18.8%) | 17727 (18.3%) | 19812 (18.0%) | 22117 (17.4%) | 25096 (16.6%) | 29718 (15.8%) | 31524 (15.2%) | 34043 (14.7%) |
| Lung Cancer | 2846 (3.8%) | 3232 (3.7%) | 3631 (3.8%) | 4173 (3.8%) | 4760 (3.7%) | 5491 (3.6%) | 6575 (3.5%) | 7206 (3.5%) | 8044 (3.5%) |
| Prostate Cancer | 27402 (36.8%) | 32038 (37.2%) | 36266 (37.5%) | 41682 (37.8%) | 49227 (38.7%) | 60186 (39.8%) | 77111 (40.9%) | 85342 (41.2%) | 95944 (41.3%) |
| **Diagnosis Cohort** |  |  |  |  |  |  |  |  |  |
| 1991-1994 | 12199 (16.4%) | 11774 (13.7%) | 11241 (11.6%) | 10810 (9.8%) | 10728 (8.4%) | 10822 (7.2%) | 12120 (6.4%) | 11683 (5.6%) | 11555 (5.0%) |
| 1995-1998 | 17436 (23.4%) | 17059 (19.8%) | 16492 (17.1%) | 16227 (14.7%) | 16412 (12.9%) | 16786 (11.1%) | 18850 (10.0%) | 18530 (8.9%) | 18683 (8.0%) |
| 1999-2002 | 43678 (58.6%) | 43512 (50.5%) | 42351 (43.8%) | 42734 (38.7%) | 43637 (34.3%) | 46277 (30.6%) | 52060 (27.6%) | 51709 (25.0%) | 52431 (22.6%) |
| 2003-2006 | 1195 (1.6%) | 13863 (16.1%) | 26603 (27.5%) | 40599 (36.8%) | 55130 (43.3%) | 59208 (39.2%) | 66531 (35.3%) | 66680 (32.2%) | 68367 (29.4%) |
| 2007-2011 |  |  |  |  | 1429 (1.1%) | 18023 (11.9%) | 38844 (20.6%) | 58642 (28.3%) | 81144 (34.9%) |
| **Census Region** |  |  |  |  |  |  |  |  |  |
| Midwest | 13891 (18.6%) | 14825 (17.2%) | 16469 (17.0%) | 18148 (16.4%) | 19940 (15.7%) | 21880 (14.5%) | 29482 (15.6%) | 30936 (14.9%) | 32554 (14.0%) |
| Northeast | 12476 (16.7%) | 14735 (17.1%) | 16863 (17.4%) | 19212 (17.4%) | 22445 (17.6%) | 31152 (20.6%) | 38225 (20.3%) | 43088 (20.8%) | 49424 (21.3%) |
| South | 14160 (19.0%) | 17596 (20.4%) | 19457 (20.1%) | 23556 (21.3%) | 28192 (22.1%) | 32831 (21.7%) | 40821 (21.7%) | 46052 (22.2%) | 51814 (22.3%) |
| West | 33981 (45.6%) | 39052 (45.3%) | 43898 (45.4%) | 49454 (44.8%) | 56759 (44.6%) | 65253 (43.2%) | 79877 (42.4%) | 87168 (42.1%) | 98388 (42.4%) |
| **Urban-Rural Status** |  |  |  |  |  |  |  |  |  |
| Metropolis | 60394 (81.1%) | 70960 (82.3%) | 79810 (82.5%) | 91220 (82.6%) | 105462 (82.8%) | 126802 (83.9%) | 159580 (84.7%) | 175752 (84.8%) | 197609 (85.1%) |
| Rural | 1718 (2.3%) | 1862 (2.2%) | 2046 (2.1%) | 2332 (2.1%) | 2640 (2.1%) | 2896 (1.9%) | 3444 (1.8%) | 3754 (1.8%) | 4102 (1.8%) |
| Urban | 12396 (16.6%) | 13386 (15.5%) | 14831 (15.3%) | 16818 (15.2%) | 19234 (15.1%) | 21418 (14.2%) | 25381 (13.5%) | 27738 (13.4%) | 30469 (13.1%) |
| **Metropolis Status** |  |  |  |  |  |  |  |  |  |
| Rural or Urban | 14114 (18.9%) | 15248 (17.7%) | 16877 (17.5%) | 19150 (17.4%) | 21874 (17.2%) | 24314 (16.1%) | 28825 (15.3%) | 31492 (15.2%) | 34571 (14.9%) |
| Metropolis | 60394 (81.1%) | 70960 (82.3%) | 79810 (82.5%) | 91220 (82.6%) | 105462 (82.8%) | 126802 (83.9%) | 159580 (84.7%) | 175752 (84.8%) | 197609 (85.1%) |
| **Original Reason for Enrollment** |  |  |  |  |  |  |  |  |  |
| Age | 68499 (91.9%) | 79149 (91.8%) | 88556 (91.6%) | 100954 (91.5%) | 116360 (91.4%) | 138358 (91.6%) | 172700 (91.7%) | 190127 (91.7%) | 213030 (91.8%) |
| Disability and End Stage Renal Disease | 26 (0.0%) | 37 (0.0%) | 41 (0.0%) | 55 (0.0%) | 62 (0.0%) | 75 (0.0%) | 102 (0.1%) | 128 (0.1%) | 141 (0.1%) |
| Disability | 5954 (8.0%) | 6989 (8.1%) | 8052 (8.3%) | 9310 (8.4%) | 10842 (8.5%) | 12591 (8.3%) | 15485 (8.2%) | 16854 (8.1%) | 18859 (8.1%) |
| End Stage Renal Disease | 29 (0.0%) | 33 (0.0%) | 38 (0.0%) | 51 (0.0%) | 72 (0.1%) | 92 (0.1%) | 118 (0.1%) | 135 (0.1%) | 150 (0.1%) |
| **Age Related Medicare Enrollment** |  |  |  |  |  |  |  |  |  |
| Not Age Related | 6009 (8.1%) | 7059 (8.2%) | 8131 (8.4%) | 9416 (8.5%) | 10976 (8.6%) | 12758 (8.4%) | 15705 (8.3%) | 17117 (8.3%) | 19150 (8.2%) |
| Age Related | 68499 (91.9%) | 79149 (91.8%) | 88556 (91.6%) | 100954 (91.5%) | 116360 (91.4%) | 138358 (91.6%) | 172700 (91.7%) | 190127 (91.7%) | 213030 (91.8%) |
| **Medicaid Eligible** | 16578 (22.2%) | 18698 (21.7%) | 20667 (21.4%) | 23357 (21.2%) | 25703 (20.2%) | 27438 (18.2%) | 27683 (14.7%) | 27611 (13.3%) | 30114 (13.0%) |
| **Charlson ≥1** | 49138 (65.9%) | 57581 (66.8%) | 64830 (67.1%) | 73821 (66.9%) | 85012 (66.8%) | 99868 (66.1%) | 123725 (65.7%) | 135520 (65.4%) | 152987 (65.9%) |
| **Depressive Disorder** | 7949 (10.7%) | 9325 (10.8%) | 10922 (11.3%) | 12609 (11.4%) | 15517 (12.2%) | 18647 (12.3%) | 23167 (12.3%) | 26378 (12.7%) | 31092 (13.4%) |
| **Anxiety Disorder** | 4877 (6.5%) | 6071 (7.0%) | 7317 (7.6%) | 8950 (8.1%) | 11458 (9.0%) | 14592 (9.7%) | 19419 (10.3%) | 22814 (11.0%) | 28640 (12.3%) |
| **Alcohol Use Disorder** | 718 (1.0%) | 790 (0.9%) | 932 (1.0%) | 1106 (1.0%) | 1416 (1.1%) | 1859 (1.2%) | 2699 (1.4%) | 3281 (1.6%) | 4174 (1.8%) |
| **Drug Use Disorder** | 395 (0.5%) | 459 (0.5%) | 584 (0.6%) | 719 (0.7%) | 928 (0.7%) | 1203 (0.8%) | 1718 (0.9%) | 2207 (1.1%) | 3297 (1.4%) |
| **Opioid Naïve** | 53491 (71.8%) | 61674 (71.5%) | 68422 (70.8%) | 76461 (69.3%) | 85718 (67.3%) | 102645 (67.9%) | 128567 (68.2%) | 142374 (68.7%) | 161347 (69.5%) |

**Supplementary Table 3.** Multivariable analysis results for Opioid Naïve Subgroup Stratified by U.S. Census Region

|  | West | Northeast | Midwest | South |
| --- | --- | --- | --- | --- |
| **Calendar Year** |  |  |  |  |
| 2008 | 0.90 (0.69, 1.16) | 0.80 (0.51, 1.25) | 1.13 (0.80, 1.60) | 1.18 (0.84, 1.66) |
| 2009 | 0.93 (0.74, 1.16) | 1.09 (0.76, 1.57) | 1.13 (0.84, 1.54) | 1.11 (0.83, 1.48) |
| 2010 | 1.01 (0.83, 1.23) | 0.92 (0.67, 1.27) | 1.26 (0.96, 1.65) | 0.88 (0.68, 1.15) |
| 2011 | 1.12 (0.94, 1.34) | 1.08 (0.82, 1.42) | 1.61 (1.27, 2.04) | 1.22 (0.98, 1.52) |
| 2012 | 1.16 (0.98, 1.36) | 1.13 (0.88, 1.45) | 1.19 (0.93, 1.50) | 0.94 (0.76, 1.15) |
| 2013 |  |  |  |  |
| 2014 | 1.02 (0.88, 1.20) | 0.88 (0.69, 1.12) | 1.18 (0.95, 1.47) | **0.78 (0.64, 0.94)** |
| 2015 | 0.92 (0.78, 1.09) | 0.83 (0.64, 1.07) | 0.80 (0.62, 1.02) | **0.58 (0.47, 0.71)** |
| 2016 | 0.85 (0.71, 1.02) | **0.71 (0.53, 0.94)** | **0.63 (0.48, 0.82)** | **0.57 (0.46, 0.71)** |
| **Years Post-Cancer Diagnosis** |  |  |  |  |
| **Age, years** |  |  |  |  |
| 66-74 | REF | REF | REF | REF |
| 75-84 | **1.20 (1.09, 1.32)** | **1.35 (1.15, 1.60)** | **1.30 (1.13, 1.51)** | 1.10 (0.97, 1.24) |
| ≥85 | **1.35 (1.21, 1.50)** | **1.56 (1.31, 1.86)** | **1.78 (1.54, 2.07)** | **1.26 (1.11, 1.44)** |
| **Cohort** |  |  |  |  |
| 1991-1994 | REF | REF | REF | REF |
| 1995-1998 | 0.98 (0.79, 1.22) | 1.09 (0.72, 1.66) | 0.97 (0.75, 1.26) | 1.35 (0.94, 1.93) |
| 1999-2002 | 0.83 (0.60, 1.14) | 0.86 (0.49, 1.52) | 1.01 (0.66, 1.52) | 1.33 (0.83, 2.13) |
| 2003-2006 | 0.75 (0.49, 1.16) | 0.75 (0.36, 1.60) | 0.95 (0.53, 1.70) | 1.51 (0.82, 2.78) |
| 2007-2011 | 0.67 (0.38, 1.17) | 0.81 (0.31, 2.08) | 1.22 (0.57, 2.62) | 2.01 (0.92, 4.36) |
| **Gender** |  |  |  |  |
| Male | REF | REF | REF | REF |
| Female | 1.16 (0.98, 1.38) | **1.34 (1.01, 1.76)** | **1.49 (1.16, 1.92)** | **1.35 (1.09, 1.68)** |
| **Race-Ethnicity** |  |  |  |  |
| Non-Hispanic White | REF | REF | REF | REF |
| Non-Hispanic Black | 1.08 (0.89, 1.31) | 1.05 (0.83, 1.33) | **1.52 (1.27, 1.82)** | 0.89 (0.76, 1.03) |
| Non-Hispanic Other | **0.47 (0.41, 0.54)** | 0.86 (0.54, 1.37) | 0.73 (0.42, 1.26) | 0.81 (0.50, 1.32) |
| Hispanic | **0.86 (0.75, 0.97)** | 1.08 (0.83, 1.40) | 1.41 (0.88, 2.27) | 0.83 (0.55, 1.25) |
| **Cancer Diagnosis** |  |  |  |  |
| Prostate | REF | REF | REF | REF |
| Breast | 1.07 (0.88, 1.30) | 0.98 (0.72, 1.33) | 0.93 (0.70, 1.24) | 0.88 (0.69, 1.12) |
| Colorectal | 1.09 (0.94, 1.27) | 0.87 (0.67, 1.13) | 0.88 (0.69, 1.12) | 0.85 (0.70, 1.05) |
| Lung | 1.16 (0.90, 1.48) | 1.07 (0.74, 1.53) | 1.17 (0.83, 1.65) | 1.13 (0.87, 1.48) |
| **Original Reason for Entitlement** |  |  |  |  |
| Age Related | REF | REF | REF | REF |
| Non-Age Related | **1.59 (1.39, 1.82)** | **1.98 (1.62, 2.43)** | **2.05 (1.72, 2.44)** | **1.35 (1.15, 1.57)** |
| **Urban-Rural Status** |  |  |  |  |
| Metropolis | REF | REF | REF | REF |
| Urban-Rural | 1.05 (0.92, 1.20) | 1.19 (0.85, 1.66) | 1.05 (0.93, 1.18) | **1.32 (1.19, 1.48)** |
| **Medicaid-Eligible** | **2.14 (1.93, 2.36)** | **1.35 (1.14, 1.60)** | **1.58 (1.36, 1.85)** | **1.77 (1.55, 2.01)** |
| **Charlson≥1** | **1.86 (1.69, 2.04)** | **1.91 (1.62, 2.25)** | **1.84 (1.61, 2.11)** | **1.80 (1.59, 2.03)** |
| **Depressive Disorder** | **1.58 (1.41, 1.76)** | **1.50 (1.26, 1.79)** | **1.51 (1.29, 1.76)** | **1.58 (1.36, 1.82)** |
| **Anxiety Disorder** | **1.38 (1.21, 1.57)** | **1.30 (1.08, 1.58)** | **1.42 (1.20, 1.68)** | **1.43 (1.22, 1.67)** |
| **Alcohol Use Disorder** | **1.64 (1.25, 2.15)** | 0.96 (0.58, 1.59) | 1.17 (0.69, 1.96) | **1.73 (1.21, 2.49)** |
| **Drug Use Disorder** | **1.91 (1.29, 2.81)** | **2.82 (1.64, 4.85)** | 1.71 (0.89, 3.28) | 1.64 (0.97, 2.78) |

**Supplementary Table 4.** Multivariable analysis results for Opioid Naïve Subgroup Stratified by U.S. Census Region

|  | West | Northeast | Midwest | South |
| --- | --- | --- | --- | --- |
| **Calendar Year** |  |  |  |  |
| 2008 | 0.87 (0.79, 0.97) | 1.03 (0.86, 1.25) | 0.90 (0.77, 1.06) | 0.86 (0.75, 0.98) |
| 2009 | 0.95 (0.87, 1.03) | 0.99 (0.85, 1.16) | 0.96 (0.84, 1.10) | 0.95 (0.86, 1.06) |
| 2010 | 1.00 (0.94, 1.07) | 1.08 (0.95, 1.23) | 1.02 (0.92, 1.14) | 0.98 (0.90, 1.08) |
| 2011 | 1.01 (0.96, 1.07) | 1.06 (0.96, 1.17) | 1.12 (1.03, 1.22) | 1.04 (0.97, 1.11) |
| 2012 | 0.96 (0.92, 1.00) | 0.99 (0.92, 1.06) | 1.03 (0.97, 1.10) | 1.00 (0.95, 1.05) |
| 2013 | REF | REF | REF | REF |
| 2014 | 0.98 (0.95, 1.02) | 0.95 (0.89, 1.02) | 0.97 (0.91, 1.03) | 0.97 (0.92, 1.01) |
| 2015 | **0.91 (0.86, 0.95)** | 0.94 (0.86, 1.02) | **0.92 (0.86, 1.00)** | **0.90 (0.85, 0.96)** |
| 2016 | **0.87 (0.82, 0.92)** | **0.87 (0.78, 0.97)** | 0.94 (0.85, 1.03) | **0.88 (0.82, 0.95)** |
| **Years Post-Cancer Diagnosis** | 1.00 (0.99, 1.02) | 1.02 (0.99, 1.05) | 1.00 (0.97, 1.03) | 1.00 (0.98, 1.03) |
| **Age, years** |  |  |  |  |
| 66-74 | REF | REF | REF | REF |
| 75-84 | **1.09 (1.05, 1.14)** | **1.13 (1.04, 1.21)** | **1.17 (1.09, 1.25)** | **1.09 (1.03, 1.14)** |
| ≥85 | **1.14 (1.09, 1.20)** | **1.27 (1.18, 1.38)** | **1.39 (1.29, 1.49)** | **1.10 (1.04, 1.17)** |
| **Cohort** |  |  |  |  |
| 1991-1994 | REF | REF | REF | REF |
| 1995-1998 | 0.94 (0.84, 1.05) | 0.90 (0.70, 1.14) | 1.01 (0.86, 1.18) | 0.96 (0.79, 1.17) |
| 1999-2002 | 1.06 (0.90, 1.25) | 1.09 (0.80, 1.48) | 1.03 (0.81, 1.32) | 1.17 (0.93, 1.47) |
| 2003-2006 | 1.05 (0.84, 1.31) | 1.13 (0.75, 1.70) | 1.05 (0.75, 1.47) | 1.21 (0.90, 1.62) |
| 2007-2011 | 1.09 (0.82, 1.45) | 1.21 (0.72, 2.04) | 1.03 (0.67, 1.60) | 1.23 (0.85, 1.78) |
| **Gender** |  |  |  |  |
| Male | REF | REF | REF | REF |
| Female | **1.25 (1.14, 1.36)** | **1.39 (1.20, 1.61)** | **1.30 (1.13, 1.49)** | **1.28 (1.15, 1.41)** |
| **Race-Ethnicity** |  |  |  |  |
| Non-Hispanic White | REF | REF | REF | REF |
| Non-Hispanic Black | **1.28 (1.16, 1.40)** | 0.97 (0.86, 1.10) | **1.52 (1.39, 1.67)** | **0.80 (0.75, 0.86)** |
| Non-Hispanic Other | **0.48 (0.44, 0.52)** | **0.59 (0.44, 0.80)** | **0.54 (0.39, 0.75)** | **0.50 (0.37, 0.68)** |
| Hispanic | **0.77 (0.72, 0.82)** | **0.68 (0.58, 0.79)** | 0.78 (0.58, 1.06) | **0.73 (0.59, 0.90)** |
| **Cancer Diagnosis** |  |  |  |  |
| Prostate | REF | REF | REF | REF |
| Breast | **1.18 (1.06, 1.30)** | 1.01 (0.86, 1.20) | 1.11 (0.95, 1.29) | 1.05 (0.94, 1.18) |
| Colorectal | **1.17 (1.08, 1.27)** | 1.04 (0.90, 1.20) | 1.07 (0.94, 1.22) | **1.10 (1.00, 1.22)** |
| Lung | **1.50 (1.35, 1.68)** | **1.31 (1.10, 1.56)** | **1.26 (1.06, 1.51)** | **1.36 (1.20, 1.53)** |
| **Original Reason for Entitlement** |  |  |  |  |
| Age Related | REF | REF | REF | REF |
| Non-Age Related | **2.16 (2.04, 2.29)** | **2.06 (1.86, 2.27)** | **1.81 (1.66, 1.98)** | **2.03 (1.90, 2.16)** |
| **Urban-Rural Status** |  |  |  |  |
| Metropolis | REF | REF | REF | REF |
| Urban-Rural | **1.26 (1.19, 1.34)** | 1.07 (0.90, 1.28) | 0.96 (0.90, 1.03) | **1.33 (1.27, 1.40)** |
| **Medicaid-Eligible** | **1.81 (1.73, 1.90)** | **1.87 (1.72, 2.03)** | **2.02 (1.87, 2.18)** | **1.92 (1.81, 2.03)** |
| **Charlson≥1** | **1.32 (1.27, 1.37)** | **1.44 (1.33, 1.56)** | **1.42 (1.33, 1.52)** | **1.30 (1.24, 1.37)** |
| **Depressive Disorder** | **1.40 (1.35, 1.45)** | **1.29 (1.20, 1.38)** | **1.33 (1.25, 1.41)** | **1.27 (1.21, 1.33)** |
| **Anxiety Disorder** | **1.24 (1.19, 1.30)** | **1.25 (1.16, 1.34)** | **1.31 (1.23, 1.40)** | **1.49 (1.42, 1.57)** |
| **Alcohol Use Disorder** | 1.07 (0.98, 1.18) | 0.91 (0.77, 1.08) | 0.96 (0.81, 1.12) | **0.79 (0.68, 0.91)** |
| **Drug Use Disorder** | **3.73 (3.44, 4.05)** | **3.75 (3.23, 4.35)** | **2.91 (2.51, 3.37)** | **4.27 (3.83, 4.76)** |

**Supplementary Table 5.** Multivariable Sensitivity analysis estimating the Adjusted Odd Ratios (aOR) and 95% Confidence Intervals of Receipt of Long-Term Opioid Therapy Stratified by U.S. Region and Prior Opioid Use in Persons with a Full Year of Observation

| **Opioid Naïve Subgroup** | | | | |
| --- | --- | --- | --- | --- |
|  | **West** | **Northeast** | **Midwest** | **South** |
| **Calendar Year** | **aOR (95% CI)** | **aOR (95% CI)** | **aOR (95% CI)** | **aOR (95% CI)** |
| 2008 | 0.89 (0.68, 1.16) | 0.83 (0.53, 1.31) | 1.05 (0.73, 1.51) | 1.24 (0.87, 1.76) |
| 2009 | 0.94 (0.74, 1.18) | 1.09 (0.76, 1.59) | 1.11 (0.81, 1.52) | 1.16 (0.87, 1.56) |
| 2010 | 1.00 (0.81, 1.22) | 0.90 (0.65, 1.26) | 1.25 (0.94, 1.65) | 0.88 (0.67, 1.16) |
| 2011 | 1.13 (0.94, 1.35) | 1.08 (0.82, 1.43) | **1.57 (1.23, 2.01)** | 1.26 (1.01, 1.58) |
| 2012 | 1.16 (0.99, 1.38) | 1.10 (0.85, 1.42) | 1.20 (0.94, 1.54) | 0.98 (0.79, 1.21) |
| 2013 | REF | REF | REF | REF |
| 2014 | 1.03 (0.88, 1.21) | 0.84 (0.66, 1.08) | 1.19 (0.95, 1.50) | **0.77 (0.63, 0.93)** |
| 2015 | 0.95 (0.80, 1.12) | 0.77 (0.59, 1.00) | 0.82 (0.63, 1.05) | **0.57 (0.46, 0.70)** |
| 2016 | 0.86 (0.72, 1.04) | **0.65 (0.49, 0.87)** | **0.65 (0.49, 0.86)** | **0.56 (0.45, 0.70)** |
| **Opioid Non-Naïve Subgroup** | | | | |
|  | **West** | **Northeast** | **Midwest** | **South** |
| **Calendar Year** | **aOR (95% CI)** | **aOR (95% CI)** | **aOR (95% CI)** | **aOR (95% CI)** |
| 2008 | **0.86 (0.78, 0.96)** | 1.04 (0.86, 1.26) | 0.90 (0.76, 1.07) | **0.86 (0.75, 0.99)** |
| 2009 | 0.95 (0.87, 1.04) | 0.97 (0.83, 1.14) | 0.97 (0.84, 1.11) | 0.95 (0.85, 1.07) |
| 2010 | 1.00 (0.93, 1.07) | 1.08 (0.95, 1.23) | 1.01 (0.90, 1.13) | 0.98 (0.89, 1.07) |
| 2011 | 1.01 (0.95, 1.07) | 1.06 (0.96, 1.17) | **1.12 (1.02, 1.22)** | 1.05 (0.98, 1.13) |
| 2012 | 0.96 (0.93, 1.00) | 1.00 (0.93, 1.08) | 1.03 (0.97, 1.10) | 1.00 (0.95, 1.05) |
| 2013 | REF | REF | REF | REF |
| 2014 | 0.98 (0.95, 1.02) | 0.96 (0.90, 1.03) | 0.98 (0.92, 1.04) | 0.96 (0.92, 1.01) |
| 2015 | **0.90 (0.86, 0.95)** | 0.94 (0.86, 1.03) | 0.94 (0.86, 1.01) | **0.90 (0.84, 0.95)** |
| 2016 | **0.87 (0.82, 0.93)** | **0.88 (0.79, 0.98)** | 0.95 (0.86, 1.05) | **0.88 (0.81, 0.95)** |

Note: Models also adjusted for Years Post-Cancer Diagnosis, Age, Diagnosis Cohort, Gender, Race and Ethnicity, Cancer Diagnosis, Original Reason for Entitlement, Urban-Rural Status, Medicaid-Eligibility, Charlson Comorbidity ≥1, Depressive Disorder, Anxiety Disorder, Alcohol Use Disorder, Drug Use Disorder. **Bolded** values indicate statistical significance at level p < 0.05.

**Supplementary Table 6.** Adjusted Odd Ratios (aOR) and 95% Confidence Intervals of Receipt of Long-Term Opioid Therapy Stratified by U.S. Region and Prior Opioid Use in Colorectal and Lung Cancer Survivors

| **Opioid Naïve Subgroup** | | | | |
| --- | --- | --- | --- | --- |
|  | **West** | **Northeast** | **Midwest** | **South** |
| **Calendar Year** | **aOR (95% CI)** | **aOR (95% CI)** | **aOR (95% CI)** | **aOR (95% CI)** |
| 2008 | 1.04 (0.60, 1.79) | 0.95 (0.38, 2.37) | 1.55 (0.78, 3.07) | 0.93 (0.45, 1.92) |
| 2009 | 1.14 (0.69, 1.87) | 1.09 (0.49, 2.39) | 1.23 (0.66, 2.29) | 1.20 (0.67, 2.14) |
| 2010 | 1.22 (0.79, 1.87) | 0.82 (0.41, 1.66) | 1.38 (0.79, 2.41) | 0.87 (0.51, 1.49) |
| 2011 | 1.13 (0.76, 1.68) | 1.22 (0.69, 2.15) | 1.45 (0.86, 2.43) | 1.10 (0.70, 1.71) |
| 2012 | 1.11 (0.76, 1.60) | 1.25 (0.75, 2.08) | 1.01 (0.60, 1.72) | 0.88 (0.58, 1.34) |
| 2013 | REF | REF | REF | REF |
| 2014 | 1.25 (0.88, 1.77) | 0.60 (0.35, 1.03) | 1.42 (0.90, 2.26) | **0.66 (0.45, 0.97)** |
| 2015 | 0.91 (0.62, 1.35) | 0.75 (0.44, 1.28) | 0.86 (0.50, 1.47) | **0.41 (0.26, 0.64)** |
| 2016 | 0.92 (0.61, 1.40) | 0.56 (0.30, 1.03) | 0.65 (0.36, 1.20) | **0.54 (0.35, 0.84)** |
| **Opioid Non-Naïve Subgroup** | | | | |
|  | **West** | **Northeast** | **Midwest** | **South** |
| **Calendar Year** | **aOR (95% CI)** | **aOR (95% CI)** | **aOR (95% CI)** | **aOR (95% CI)** |
| 2008 | **0.59 (0.47, 0.74)** | **0.55 (0.35, 0.87)** | **0.64 (0.46, 0.88)** | 0.80 (0.62, 1.03) |
| 2009 | **0.67 (0.55, 0.80)** | **0.58 (0.40, 0.85)** | **0.67 (0.52, 0.88)** | **0.80 (0.65, 0.98)** |
| 2010 | **0.73 (0.63, 0.85)** | **0.67 (0.49, 0.91)** | 0.81 (0.65, 1.00) | 0.86 (0.73, 1.02) |
| 2011 | **0.82 (0.73, 0.92)** | 0.82 (0.65, 1.04) | 0.97 (0.82, 1.15) | 0.99 (0.87, 1.12) |
| 2012 | **0.88 (0.81, 0.97)** | **0.82 (0.68, 0.98)** | 1.03 (0.90, 1.18) | 1.01 (0.92, 1.11) |
| 2013 | REF | REF | REF | REF |
| 2014 | **1.10 (1.01, 1.20)** | 1.11 (0.94, 1.31) | 1.03 (0.91, 1.17) | 0.98 (0.90, 1.08) |
| 2015 | 1.11 (1.00, 1.23) | 1.14 (0.92, 1.41) | 0.99 (0.85, 1.15) | 0.92 (0.82, 1.03) |
| 2016 | 1.09 (0.96, 1.24) | 1.25 (0.94, 1.65) | 0.99 (0.82, 1.20) | 0.95 (0.82, 1.09) |

Note: **Bolded** values indicate statistical significance at level of p<0.05.

**Supplementary Table 7.** Adjusted Odd Ratios (aOR) and 95% Confidence Intervals of Receipt of Long-term Opioid Therapy Stratified by Cancer Diagnosis

|  | **Breast Cancer Survivors** | **Colorectal Cancer Survivors** | **Lung Cancer Survivors** | **Prostate Cancer Survivors** |
| --- | --- | --- | --- | --- |
| **Calendar Year** | **aOR (95% CI)** | **aOR (95% CI)** | **aOR (95% CI)** | **aOR (95% CI)** |
| 2008 | **0.91 (0.84, 0.99)** | **0.81 (0.71, 0.93)** | 1.00 (0.79, 1.27) | **0.85 (0.76, 0.94)** |
| 2009 | **0.91 (0.85, 0.98)** | **0.83 (0.74, 0.93)** | 1.05 (0.86, 1.27) | **0.84 (0.77, 0.92)** |
| 2010 | **0.92 (0.87, 0.97)** | **0.89 (0.81, 0.98)** | 1.06 (0.91, 1.24) | **0.89 (0.83, 0.95)** |
| 2011 | 1.02 (0.98, 1.06) | 0.97 (0.90, 1.04) | 1.07 (0.95, 1.21) | 0.96 (0.91, 1.01) |
| 2012 | 0.99 (0.96, 1.02) | 0.98 (0.93, 1.03) | 0.97 (0.89, 1.07) | 0.99 (0.95, 1.03) |
| 2013 | REF | REF | REF | REF |
| 2014 | 0.99 (0.96, 1.02) | 1.01 (0.96, 1.06) | 1.04 (0.96, 1.13) | 1.02 (0.98, 1.06) |
| 2015 | 0.96 (0.92, 0.99) | 0.97 (0.91, 1.04) | 0.97 (0.87, 1.08) | 0.97 (0.93, 1.02) |
| 2016 | 0.95 (0.90, 1.00) | 1.04 (0.96, 1.14) | 0.98 (0.85, 1.12) | 1.04 (0.98, 1.10) |
| **Years Post-Cancer Diagnosis** | 1.00 (0.98, 1.01) | 0.98 (0.95, 1.00) | 0.98 (0.94, 1.02) | 0.99 (0.97, 1.01) |
| **Age, years** |  |  |  |  |
| 66-74 | REF | REF | REF | REF |
| 75-84 | **1.16 (1.12, 1.20)** | 1.02 (0.97, 1.08) | **0.84 (0.77, 0.91)** | 0.99 (0.96, 1.03) |
| ≥85 | **1.19 (1.16, 1.23)** | 1.04 (0.98, 1.09) | **0.85 (0.78, 0.92)** | 1.03 (0.99, 1.07) |
| **Cohort** |  |  |  |  |
| 1991-1994 | REF | REF | REF | REF |
| 1995-1998 | 0.93 (0.85, 1.03) | 0.90 (0.76, 1.06) | 0.97 (0.69, 1.36) | **0.85 (0.74, 0.96)** |
| 1999-2002 | 1.03 (0.90, 1.18) | 0.88 (0.70, 1.11) | 0.88 (0.58, 1.34) | 0.86 (0.72, 1.02) |
| 2003-2006 | 1.02 (0.85, 1.23) | 0.90 (0.66, 1.22) | 0.99 (0.58, 1.69) | 0.87 (0.69, 1.10) |
| 2007-2011 | 1.13 (0.89, 1.44) | 0.92 (0.62, 1.37) | 1.10 (0.56, 2.14) | 0.89 (0.67, 1.19) |
| **Gender** |  |  |  |  |
| Male | REF | REF | REF | REF |
| Female | **-** | **1.41 (1.33, 1.50)** | **1.31 (1.19, 1.43)** | **-** |
| **Race-Ethnicity** |  |  |  |  |
| Non-Hispanic White | REF | REF | REF | REF |
| Non-Hispanic Black | 1.02 (0.96, 1.09) | 1.05 (0.96, 1.15) | 1.01 (0.86, 1.18) | **1.26 (1.18, 1.34)** |
| Non-Hispanic Other | **0.36 (0.32, 0.40)** | **0.31 (0.26, 0.37)** | **0.36 (0.26, 0.48)** | **0.50 (0.44, 0.56)** |
| Hispanic | **0.75 (0.69, 0.81)** | **0.74 (0.65, 0.83)** | 0.82 (0.66, 1.03) | **0.79 (0.73, 0.86)** |
| **Census Region** |  |  |  |  |
| West |  | REF | REF |  |
| Northeast | **0.61 (0.58, 0.64)** | **0.63 (0.58, 0.69)** | **0.60 (0.53, 0.69)** | **0.62 (0.58, 0.67)** |
| Midwest | **1.11 (1.06, 1.16)** | 1.07 (0.98, 1.16) | 0.93 (0.81, 1.07) | **1.15 (1.09, 1.22)** |
| South | **1.06 (1.02, 1.11)** | 1.05 (0.98, 1.12) | 1.00 (0.90, 1.12) | 1.05 (1.00, 1.11) |
| **Original Reason for Entitlement** |  |  |  |  |
| Age Related | REF | REF | REF | REF |
| Non-Age Related | **2.57 (2.46, 2.69)** | **2.13 (1.98, 2.29)** | **2.12 (1.91, 2.35)** | **2.73 (2.59, 2.88)** |
| **Urban-Rural Status** |  |  |  |  |
| Metropolis | REF | REF | REF | REF |
| Urban-Rural | **1.16 (1.11, 1.21)** | **1.16 (1.08, 1.24)** | **1.30 (1.17, 1.45)** | **1.26 (1.20, 1.33)** |
| **Medicaid-Eligible** | **1.72 (1.66, 1.78)** | **1.84 (1.74, 1.94)** | **1.91 (1.74, 2.09)** | **2.09 (1.99, 2.19)** |
| **Charlson≥1** | **1.22 (1.19, 1.24)** | **1.21 (1.16, 1.26)** | **1.24 (1.14, 1.35)** | **1.26 (1.22, 1.29)** |
| **Depressive Disorder** | **1.19 (1.16, 1.22)** | **1.11 (1.06, 1.16)** | 1.06 (0.99, 1.14) | **1.20 (1.15, 1.25)** |
| **Anxiety Disorder** | **1.12 (1.10, 1.15)** | **1.13 (1.08, 1.19)** | **1.26 (1.17, 1.35)** | **1.21 (1.16, 1.27)** |
| **Alcohol Use Disorder** | 0.98 (0.91, 1.06) | 1.05 (0.93, 1.18) | 1.01 (0.83, 1.22) | 1.04 (0.96, 1.12) |
| **Drug Use Disorder** | **1.43 (1.34, 1.53)** | **1.60 (1.44, 1.78)** | **1.46 (1.26, 1.69)** | **1.54 (1.41, 1.68)** |
| **Opioid Naïve** | **0.29 (0.29, 0.30)** | **0.24 (0.23, 0.24)** | **0.20 (0.19, 0.21)** | **0.25 (0.24, 0.25)** |

Note: **Bolded** values indicate statistical significance at level of p<0.05.
